# Mapping abiotic stresses for rice in Africa: Drought, cold, iron toxicity, salinity and sodicity

**DOI:** 10.1016/j.fcr.2018.01.016

**Published:** 2018-04-15

**Authors:** P.A.J. van Oort

**Affiliations:** a Africa Rice Center (AfricaRice), 01 B.P. 2551, Bouaké, Cote d’Ivoire; b Crop & Weed Ecology Group, Centre for Crop Systems Analysis, Wageningen University, P.O. Box 430, 6700 AK, Wageningen, The Netherlands

**Keywords:** GIS, ORYZA2000, HWSD, Crop maps, Uncertainty

## Abstract

Maps of abiotic stresses for rice can be useful for (1) prioritizing research and (2) identifying stress hotspots, for directing technologies and varieties to those areas where they are most needed. Large-scale maps of stresses are not available for Africa. This paper considers four abiotic stresses relevant for rice in Africa (drought, cold, iron toxicity and salinity/sodicity). Maps showing hotspots of the stresses, the countries most affected and total potentially affected area are presented. In terms of relative importance, the study identified drought as the most important stress (33% of rice area potentially affected), followed by iron toxicity (12%) and then cold (7%) and salinity/sodicity (2%). Hotspots for iron toxicity, cold and salinity are identified. For drought, local variation along the hydromorphic zone was a stronger determinant than larger-scale climatic variation, therefore mapping of drought based on climatic zones has only limited value. Uncertainties in the mappings are discussed.

## Highlights

• Hotspots of drought, cold, iron toxicity salinity/sodicity stress occurrence for rice in Africa.
• Maps for targeted distribution of tolerant varieties.
• Drought most important stress (33% of rice area) then iron toxicity (12%).
• Risk of cold/salinity/sodicity in 7–2% of Africa’s rice area.

## 1 Introduction

Maps of crop stresses can be used for research prioritization (Waddington et al., 2010). They can be used to focus research and development activities on the most important stresses. And they can be used to target dissemination of solutions for specific stresses. For example, for distributing varieties tolerant to iron toxicity specifically to those areas where iron toxicity is widely present. This paper focusses on four abiotic stresses in rice (*Oryza* spp.): drought, cold, iron toxicity and salinity. These four stresses were selected because (1) they are known to be important for rice (Balasubramanian et al., 2007; Diagne et al., 2013b) and (2) they are relevant in the context of a large breeding programme focused particularly on these four stresses, the Stress-Tolerant Rice for Africa and South Asia (STRASA) project (the fifth STRASA stress, flooding, is not mapped here).

There have been limited efforts to develop continent-wide maps of rice stresses in Africa. Diagne et al. (2013b) and Waddington et al. (2010) used surveys to identify major stresses. The most important constraints identified by the experts consulted by Waddington et al. (2010) were those of fertiliser supply/soil fertility, drought/water management and problems with weeds. Diagne et al. (2013b) report major constraints identified through farmer surveys for four rice-growing environments in Africa (irrigated, rainfed upland, rainfed lowland and “other”). Weeds, rodents and birds, and diseases were reported as the main constraints. The emphasis of Diagne et al. (2013b) and Waddington et al. (2010) is more on identifying the most important constraints than on mapping them.

Two other studies with continent-wide coverage are more “spatially explicit”. Haefele et al. (2014) present global maps and area estimates of soil quality classes and constraints for rice. For rice in Africa, they identify low soil fertility as the main soil constraint (37.6% of all rice area in Africa, i.e. 3.94 Mha of a total rice area of 10.47 Mha), followed by drought (19.0%) and aluminium toxicity (18.8%); the latter is strongly linked to soil phosphate-fixation, causing phosphorus (P) deficiencies for rice and other crops. The drought analysis by Haefele et al. (2014) is based on soil water-holding capacity only, not on climatic data. Soils with low water-holding capacity were considered drought-prone. However, in humid climates or in areas with high groundwater levels, a low water-holding capacity need not be a problem. If there is no rain for a long time during the growing season then, no matter what the water-holding capacity, crops will experience drought. In this sense rice is more vulnerable than most other crops, because it has, with its shallow rooting system (20–40 cm), access to only a small volume of soil. An analysis of drought risk would benefit from taking into consideration rainfall and groundwater-table depth. The second “spatially explicit” continent-wide study was on major weeds in rice. According to Rodenburg et al. (2016), an estimated 1.34 Mha of rainfed rice is infested with at least one of the weeds *Striga asiatica*, *S. aspera* and *S. hermonthica* in rainfed uplands, and *Rhamphicarpa fistulosa* in rainfed lowlands. All four studies cited above discuss uncertainties associated with data and their use, which are large, and include uncertainties in the rice maps used.

Thus, for the four abiotic stresses of drought, cold, iron toxicity and salinity, few or no maps have been developed at the continental scale for rice in Africa. Only drought has been mapped to a limited extent by Haefele et al. (2014). Meanwhile, tolerant varieties for these abiotic stresses are in different stages of development.

Since salinity and sodicity are frequently found in the same places, in this paper a broader definition of salinity is adopted, including also sodic (also called alkaline) soils.

The objective of this paper was to map the four stresses, drought, cold, iron toxicity and salinity/sodicity. For each stress, the area potentially affected was estimated per country. The most affected countries are highlighted in tables and maps. More maps are provided online to allow readers to zoom in and identify hotspots for each stress.

## 2 Materials and methods

### 2.1 General approach

The general approach was to overlay a rice crop map with a “stressor” map to identify those areas with both rice and the “stressor”. For each stress, uncertainties were identified via an extensive literature review and these uncertainties were then quantified using different input datasets. Owing to these uncertainties, we speak of “potentially affected” areas. For each stress, the area potentially affected is estimated.

### 2.2 Spatial datasets

#### 2.2.1 Crop maps

Using three crop maps gives a sense of the uncertainty in estimating potentially affected areas because of uncertainty about where rice is grown. All three maps of rice harvested area (ha) have a 0.083° spatial resolution (approximately 9 × 9 km):

• SPAM2005 data for rice, downloaded from http://harvestchoice.org/data/rice_h (You et al., 2014a, You et al., 2014b)
• MIRCA2000 data for rice, downloaded from http://www2.uni-frankfurt.de/45218023/MIRCA (Portmann et al., 2010)
• GAEZv3 data for rice, downloaded from www.gaez.iiasa.ac.at/ (Fischer et al., 2013).

Differences between these maps have been investigated by Anderson et al. (2015). All three make a distinction between rainfed and irrigated crops and provide separate maps for those. Rodenburg et al. (2016) note that these maps indicate a number of African countries with little or no data for rainfed rice while in reality we know there is a substantial rainfed rice area. This is also illustrated in two examples in the Appendix (§A.1), which clearly show that these maps are, for rice in Africa, too uncertain in terms of differentiating between where irrigated and rainfed rice are located. Consequently, I calculated the areas of rainfed lowland, rainfed upland, irrigated and mangrove rice by multiplying the mapped total rice area (SPAM2005, MIRCA2000, GAEZv3) by country fractions of rainfed lowland/upland and irrigated land calculated from country data reported by Diagne et al. (2013a). A drawback of this approach is that we remain less certain about the spatial distribution of these rice-growing environments within the countries, but it avoids the obvious gross allocation errors between irrigated and rainfed in the three crop maps.

#### 2.2.2 Harmonised World Soil Database (HWSD)

Risk of iron toxicity and salinity was mapped using the Harmonised World Soil Database (HWSD). The HWSD is a course-scale map, 1:5,000,000 (FAO et al., 2012). It has 16,327 unique spatial mapping units (SMUs). Each SMU contains 1–10 (median 3) non-georeferenced soil units (Haefele et al., 2014). The online available raster version at 0.0083° resolution (approximately 0.9 × 0.9 km) was used. First, the share of iron/saline soil units was calculated for each SMU. This high-resolution iron/salinity map was aggregated (spatial average) to the same spatial resolution as the three crop maps and overlaid with the crop maps to identify potentially affected areas, i.e. those with both iron or salts and rice.

#### 2.2.3 Climate zonation maps

The Köppen–Geiger climate zone map (Kottek et al., 2006) was used to spatially extrapolate site-based estimates of drought and cold stress. A key issue is whether climate zone maps are suitable for such extrapolation. Extrapolation becomes impossible with no simulation sites inside a climate zone. Extrapolation becomes highly uncertain with just one or few simulation sites inside. Extrapolation also becomes uncertain when within-zone (short scale) variation is larger than between-zone variation.

Van Wart et al. (2013) review 12 climate zone maps. Of these, six have fewer zones (9–25 zones) than the Köppen–Geiger (which has 31 zones) and five have more zones (74–265). A test (not shown) revealed that with 74 or more zones, there would be many zones with no or few simulation sites inside, which can therefore not be used for spatial extrapolation. Therefore, the five zonation maps with high numbers of zones were not considered suitable for the objective of presenting a map of drought or cold risk for the whole of Africa. Of the remaining seven maps with fewer zones (6–31), the Köppen–Geiger climate zonation has the largest number of zones (31), therefore the Köppen–Geiger climate zone map was considered most suitable for full spatial coverage at the highest viable spatial resolution.

### 2.3 ORYZA2000 model

Drought and cold stress were simulated with the model ORYZA2000v2n14. This version is based on ORYZA2000v2n13s14 as documented by van Oort et al. (2015a). This model includes recent updates on modelling heat, cold and phenology documented by van Oort et al. (2015a). A common set of sites, weather data, sowing dates and phenological parameters were used, which are documented in this section. More details on how drought and cold were simulated are provided in the following sections.

#### 2.3.1 Site and season selection

Sites for drought and cold were selected with a protocol described by Van Wart et al. (2013). Sites were selected such that they together covered the major climatic zones and crop regions within a country. Countries were chosen such that these together represent the different agro-ecologies and major rice regions of the African continent, i.e. East, West and North Africa, irrigated and rainfed, and lowlands and highlands. In total, 19 countries were selected (West 11, North 1, East 7), with 53 irrigated sites and 52 rainfed sites.

#### 2.3.2 Weather data

A site was defined as a weather-station point. For each point, the associated pixel of the AgMERRA weather dataset was identified. The AgMERRA dataset (Ruane et al., 2015) contains daily weather data from 1980 to 2010 at a 0.25° × 0.25° resolution (∼28 × 28 km). Potential and water-limited yields (Bouman et al., 2001) were simulated for irrigated sites (1998–2002, five years) and rainfed rice (1996–2004, nine years). For irrigated sites, fewer simulations were needed to obtain robust long-term average stress indices because interannual variability in yields is less, due to smaller climatic risks.

#### 2.3.3 Sowing dates and phenology

Sowing dates and crop duration were derived from the RiceAtlas (Laborte et al., 2017) and cross-checked with additional data when initial simulations showed unrealistically low simulated yields. Temperature sums for different developmental stages were calibrated assuming a base temperature (TBD) of 14 °C and an optimal temperature (TOD) of 31 °C and no delay in development above the optimum temperature (van Oort et al., 2011; Zhang et al., 2016). The calibrated average duration from flowering to maturity was 30 days for all sites (Vergara and Chang, 1985). The simulated duration from panicle initiation to flowering was 0.35 of the duration from emergence to flowering and the number of days from emergence to panicle initiation as 0.65 of the duration from emergence to flowering (Bouman et al., 2001).

### 2.4 Drought

#### 2.4.1 Soil parameters

Rainfed rice yields depend strongly on groundwater depth and percolation losses (Boling et al., 2007; Bouman et al., 1994, Bouman et al., 2007; Wopereis et al., 1994). Rainfed lowland generally has high groundwater levels and low percolation rates; rainfed upland generally has freely draining soils, i.e. with deep groundwater levels and high percolation rates. As one moves from the highest landscape positions (upland rice) to the lowest (lowland rice), one moves through the hydromorphic slopes with increasingly shallow groundwater depth (Schmitter et al., 2015). No large-scale high-resolution groundwater datasets are available for Africa. Therefore, rainfed rice yields were simulated for two contrasting positions along this continuum:

• a typical lowland soil (clayey, high groundwater level, bunds) and
• a typical upland soil (sandy, free-draining, no bunds).

Soil parameters are listed in the Appendix (§A.2).

#### 2.4.2 Sowing dates

The start of the wet season and drought during the season can vary strongly from year to year depending on rainfall patterns. Crop exposure to drought therefore depends strongly on sowing date. Accurate simulation depends on accurate estimation of the sowing date. To mimic farmers’ decision-making we simulated sowing dates such that these would coincide with the start of the wet season. A similar rainfall rule to that developed by Wolf et al. (2015) was used: the simulated sowing day was the first possible day in which cumulative rainfall over the previous 7 days was at least 20 mm, within a sowing window of ±30 days around the average sowing date of a particular site.

#### 2.4.3 Risk indicator and area potentially affected

Drought risk was calculated as Yw/Yp, where Yw is the water-limited yield and Yp the potential yield (Bouman et al., 2001). Thus Yw/Yp = 1 means no drought stress and Yw/Yp = 0 means complete crop failure due to drought. For each site, the average Yw/Yp value was calculated for both lowland and upland. Year-on-year variation in drought risk is important (e.g. in respect of years of total crop failure); however, the 9-year minima were strongly correlated with the 9-year averages (lowland: *R*^2^ = 0.78; upland *R*^2^ = 0.60), therefore only averages are reported here.

### 2.5 Cold

#### 2.5.1 Cold-induced sterility

**Fig. 1:**
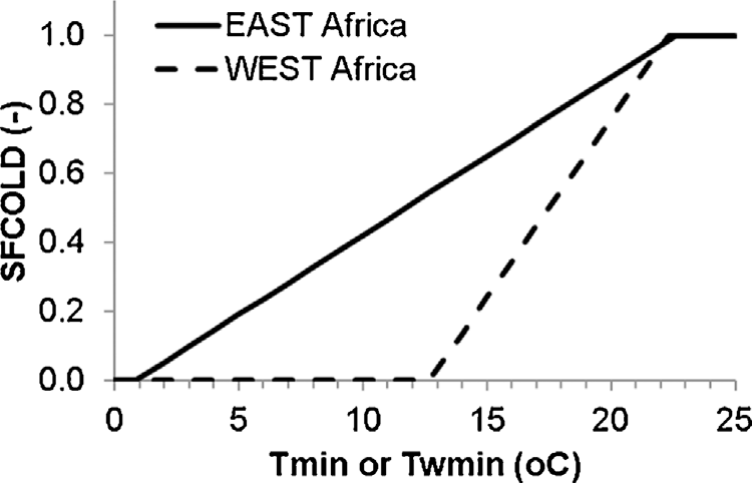
**Cold sterility functions used in the current study, based on**Dingkuhn et al. (2015). On the y-axis, SFCOLD is the spikelet fertility caused by cold; on the x-axis is the minimum air temperature T_min_. T_wmin_ is the minimum water temperature. The model described by van Oort et al. (2015a) distinguishes between two cold-sensitive phases: in the first phase T_wmin_ is used; in the second phase T_min_ is used.

The effects of cold on rice are still poorly understood. Cold has two main effects: (1) it leads to a long growing season, which may prohibit growth of two or three crops per year; and (2) it can lead to cold-induced sterility. Dingkuhn et al. (2015) measured cold-induced sterility for three varieties in Senegal and Madagascar, and found that, at the same minimum water temperature from booting to heading, the varieties grown in Senegal showed much greater sterility. The cause of this difference is not yet fully understood, the most plausible hypothesis is that a process of acclimation is occurring in Madagascar, where rice plants exposed longer to cold would be more cold tolerant (Dingkuhn et al., 2017). At the time of writing, the best possible approximation was to simulate cold sterility with East and West Africa equations. These two regions have different cold regimes: in West Africa, cold occurs mainly in irrigated rice systems in the Sahel zone with large diurnal temperature amplitudes, requiring tropical varieties adapted to high daytime temperatures, which may be less adapted to night-time cold. Whereas in East Africa cold occurs in the highlands with prolonged cold and smaller diurnal temperature amplitudes, and varieties adapted to this cooler climate may be better adapted to cold. Fig. 1 shows the cold sterility functions for variety IR64 derived from Dingkuhn et al. (2015). These were incorporated into ORYZA2000, and cold sterility was subsequently simulated following methods described by van Oort et al. (2015a). Depending on country, either the West Africa equation (Eq. (1a)) or the East Africa equation (Eq. (1b)) was used. The model described by van Oort et al. (2015a) distinguishes between two cold-sensitive phases. I the first phase spikelet fertility SFCOLD (Eqs. (1a) and (1b)) is predicted from the minimum water temperature (*T* = *T_w_*_min_). In the second phase sterility is predicted using minimum air temperature (*T* = *T*_min_). For the North African site in the Nile delta, the East Africa equation was used.

(1a) $$SFCOLD=\begin{array}{cc} \text{max}\left(0,\text{min}\left(1,1-\left(2.32-0.104\times T\right)\right)\right) & \mathrm{West}\;\;\mathrm{Africa} \end{array}$$

(1b) $$SFCOLD=\begin{array}{cc} \text{max}\left(0,\text{min}\left(1,1-\left(1.04-0.046\times T\right)\right)\right) & \mathrm{East}\;\;\mathrm{Africa} \end{array}$$

#### 2.5.2 Risk indicator and area potentially affected

The damage done by cold sterility can be strongly influenced by the number of spikelets and by photosynthesis during the grain-filling phase (van Oort et al., 2015a). Normally about 5–15% of the spikelets remain unfilled simply because there are more spikelets than the plant can fill given the amount of photosynthesis during the grain-filling phase. Cold sterility is more severe for a sink-limited crop, i.e. a crop with few spikelets, which can in turn be caused by low soil fertility (Yoshida et al., 2006). Spikelet fertility determined by cold (SFCOLD) was used as the cold-stress indicator. Cold-sensitive areas were marked as those having an average cold sterility of >20% or >50%.

### 2.6 Iron toxicity

#### 2.6.1 Rainfed lowland rice

Iron is abundant in the earth’s crust. Normally, it occurs as Fe^3+^ (hydro)oxides. When flooded for a few days or more, Fe^3+^ is reduced to Fe^2+^, which at high concentrations is toxic to plants (Becker and Asch, 2005). Iron toxicity is therefore commonly found in rice and rarely in other crops because rice can grow, unlike most other crops, in prolonged flooded conditions. Iron toxicity is also a problem of high Fe^2+^ concentration relative to concentrations of other positively charged atoms and is therefore more often found in highly weathered soils in the tropics, which have relatively high Fe^3+^ and Al^3+^ concentrations (most other positively charged elements are more easily dissolved and have therefore already been leached). In the current study, the following assumptions were made:

1. No risk of iron toxicity in irrigated rice: in most cases drainage is possible to get rid of excess Fe^2+^;
2. No risk of iron toxicity in rainfed uplands: these rarely have prolonged flooded conditions, and so dissolved Fe^2+^ runs off;
3. Risk of iron-toxicity in rainfed lowlands on “iron-rich soils”: prolonged flooded conditions, often poor drainage and iron-rich run-on from higher landscape positions.

A justification for this focus on rainfed lowlands also follows from considering the situation of abundant iron-rich Ferralsols in the Brazilian savannah (Cerrado), where rice is also grown (Heinemann et al., 2015). It is grown there almost exclusively as upland rice in the wet season and iron toxicity is not considered a major problem (Alexandre Heinemann, Personal communication). It is really the combination of prolonged flooded conditions and poor drainage on “iron-rich soils” that creates a risk of iron toxicity.

#### 2.6.2 Iron-rich soil types

Becker and Asch (2005) define three environments in which iron toxicity occurs: (1) in the coastal plains and river deltas on young acid-sulphate soils; (2) in marshes, highland swamps, on clayey Ultisols and Histosols; and (3) in inland-valley swamps on sandy valley-bottom soils. Soil types with iron-toxicity risk were identified based from the literature (Audebert and Fofana, 2009; Audebert et al., 2006; Becker and Asch, 2005; Chérif et al., 2009; Genon et al., 1994; Howeler, 1973; Jugsujinda and Patrick, 1993; Prade et al., 1993; Sahrawat, 2004; Sikirou et al., 2015, Sikirou et al., 2016). Iron toxicity in rice has been reported on the following soils

• Oxisols (USDA soil taxonomy) = Ferralsols (WRB & FAO soil taxonomy^1^)
• Ultisols (USDA soil taxonomy) = mostly Acrisols; also Alisols or Nitisols (WRB & FAO)
• Alfisols (USDA soil taxonomy) = Luvisols or Lixisols, and some Nitosols (WRB & FAO)
• Acid-sulphate soils = Thionic soils.

These soils differ in their iron content, therefore only those soils with high iron content (“ferric” soil property) were included, see the Appendix (§A.3) for the exact list of soils included. Surprisingly, no iron toxicity has been reported for Plinthosols, which also have high iron content. Plinthosols are classified as “mineral soils conditioned by a wet (sub)tropical climate” (Driessen and Dudal, 1989; Dudal and Driessen, 1991); this is the same “habitat” that has iron-toxicity-risk Ferralsols, Acrisols, Alisols, Nitisols and Lixisols. There are Acrisols, Gleysols, Luvisols, Lixisols and Alisols with plinthite in their soil profiles. It is possible that the presence of plinthite has been implicitly considered a risk factor but not reported as such. To address this uncertainty on Plinthosols, two iron-toxicity maps were generated, one with and one without Plinthosols.

#### 2.6.3 Risk indicator and area potentially affected

The area potentially affected by iron toxicity was calculated as the fraction “iron-rich soils” (HWSD; with/without plinthosols) multiplied by the area of rice (3 crop maps) multiplied by the national fraction rainfed lowland (Diagne et al., 2013a).

### 2.7 Salinity and sodicity

#### 2.7.1 Threshold values and soil types

Salinity often coincides with sodicity and breeders breeding for tolerance to salinity often simultaneously select for tolerance to sodicity. Salinity/sodicity occurs in mangrove areas. Inland salinity is found in places with saline/sodic parent material plus potential evapotranspiration exceeding rainfall, i.e. quite dry regions. Where rivers cross such dry regions, irrigated rice on saline/sodic soils can be found. Two maps were made based on HWSD, one showing only saline soils, the other showing saline and sodic soils

• Solonchaks + Salic Fluvisols (SCFLs, saline)
• Solonchaks + Salic Fluvisols (saline) + Solonetz (SCFLsSN, saline or sodic).

The Appendix (§A.4) presents a closer analysis of electrical conductivity (EC) and exchangeable sodium percentage (ESP) values of these soil types as contained in the HWSD soil map.

Additional to the HWSD salinity map, country estimates of mangrove rice area from Diagne et al. (2013a) are presented. Diagne et al. (2013a) used total rice area per country from FAOSTAT and multiplied these by fractions of different rice-growing environments at the national level determined from household surveys. Their rice-growing environment called “other” contains mainly mangrove rice but also deep-water and floating rice, for example inland along rivers such as the Niger River. Mangrove rice area estimates by Diagne et al. (2013a) cannot be compared one-to-one with saline/sodic rice area estimate (HWSD × crop maps) because (1) the crop maps used with HWSD show rice area around 2000–2005, while Diagne et al. (2013a) used FAOSTAT rice areas in 2012. Substantial rice area expansion has taken place since 2000–2005, so mangrove rice area tends to be larger in Diagne et al’s dataset than in the HWSD × crop maps overlay; (2) the HWSD × crop maps show salinity/sodicity both inland and along the coasts, whereas mangrove is hardly found far inland; and (3) if we were to classify all “other” rice environments in Diagne et al. (2013a) as mangrove, we may overestimate the mangrove rice area. With these precautions in mind, we can still use mangrove rice area estimates from Diagne et al. (2013a) for a consistency check, to cross-check whether countries with large areas of mangrove rice are also identified as such by the HWSD.

#### 2.7.2 Risk indicator and area potentially affected

The area potentially affected by salinity/sodicity was calculated as the fraction of saline/sodic soils (HWSD; with/without sodic soils) multiplied by the area of rice (3 crop maps). The lower estimate of area potentially affected was calculated as the area of rice on saline soils. The upper estimate was calculated as the area of rice on saline plus sodic soils.

### 2.8 Visualisation in Google Earth

Visualisation in Google Earth allows readers to zoom in to identify and geo-reference stress hotspots, and it allows readers to validate the results presented in this study. For each stress, Google Earth maps are available as supplementary information. For drought and cold, Köppen–Geiger climate zones (Kottek et al., 2006, http://koeppen-geiger.vu-wien.ac.at) are shown with an extra legend inset with drought/cold stress labels. For iron toxicity and salinity/sodicity, gridded maps (9 × 9 km resolution) were generated with the plotKML package in R (Hengl et al., 2015).

## 3 Results

### 3.1 Crop areas

Table 1, Table 2 list total crop areas according to the three crop maps and fraction of different rice-growing environments according to Balasubramanian et al. (2007) and Diagne et al. (2013a). A distinction between sub-Sahara Africa (SSA) and North Africa is relevant here, because in North Africa (mostly Egypt) almost all rice is irrigated, whereas in SSA most rice is rainfed. This distinction is relevant for the stresses that occur in rainfed rice areas. The fractions of different rice-growing environments reported by Balasubramanian et al. (2007) and Diagne et al. (2013a) are similar, which supports the confidence in these data.

**Table 1:** Total rice area (Mha) according to the three crop maps used in this study.

Map	SSA	North Africa	Africa
MIRCA2000	6.114	0.658	6.772
SPAM2005	8.037	0.652	8.689
GAEZv3	6.939	0.627	7.565
Average	7.030	0.646	7.675

**Table 2:** Fraction of total rice area in the different rice-growing environments.

Environment	SSA^a^	North Africa^a^	Africa total^a^	Africa total^b^
Irrigated	0.22	0.89	0.26	0.20
Rainfed lowland	0.40	0.03	0.38	0.33
Rainfed upland	0.34	0.03	0.32	0.38
Other^c^	0.04	0.05	0.04	0.09

a Based on Diagne et al. (2013a).

b Based on Balasubramanian et al. (2007).

c “Other” is mainly mangrove rice, plus some deep-water rice along major rivers.

### 3.2 Drought

**Fig. 2:**
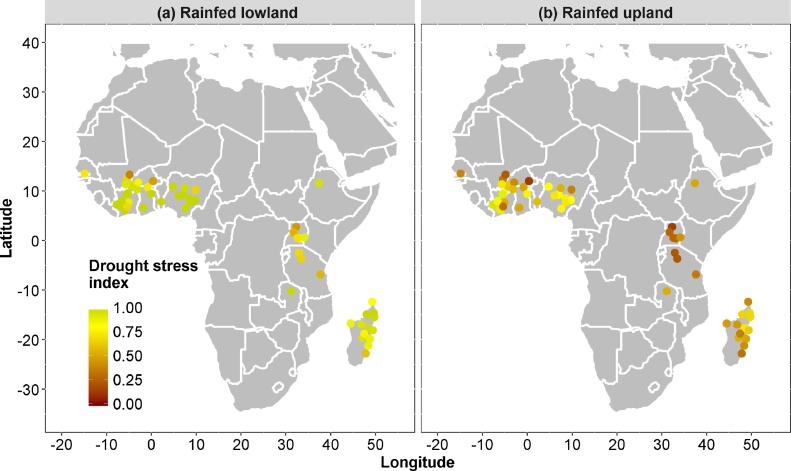
**Simulated drought stress.** Left: rainfed lowland. Right: rainfed upland. Dots are sites for which drought stress was simulated using 9 years of weather data for each dot. The drought stress index is Yw/Yp, i.e. the water-limited yield (with water stress) divided by the potential yield (without drought stress); a low value means severe loss due to drought.

**Fig. 3:**
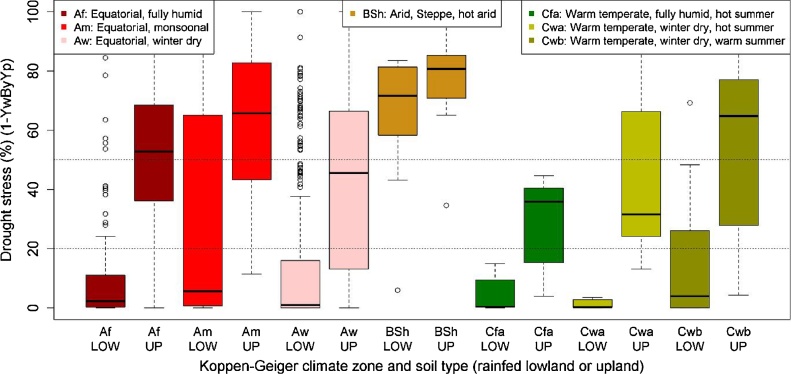
**Drought stress in the Köppen–Geiger climate zones: boxplots.** Drought stress here is expressed as 100 × (1 − Yw/Yp). Box plots are based on annual multi-site simulations, with 1 or 2 seasons. The black horizontal line in each box is the median. Top and bottom of the box are the 25th and 75th percentiles (Q1 and Q3). The “dots” at the end of the boxplot represent outliers: Outliers are points with: less than Q1 − (1.5 × IQR) or greater than Q3 + (1.5 × IQR), with IQR = Q3 − Q1. Box colours are consistent with the Köppen–Geiger colour scheme (Kottek et al., 2006). LOW = lowland; UP = upland.

Fig. 2 shows that simulated drought stress occurs throughout the continent. Drought stress is more severe in some places (West Africa north of 10°N, East Africa mainland) and less in others (West Africa south of 10°N, northern part of Madagascar). Fig. 2, Fig. 3 show (1) severe drought risk in all upland soils in all climate zones, (2) limited drought risk in lowlands in most climate zones and (3) moderate–severe risk in a few climate zones (Am, BSh). There was one site (out of 52 rainfed sites) with rainfed rice in the BSh (hot arid steppe) climate zone with severe drought risk. While this site does not have a full-blown irrigation system with irrigation and drainage canals, farmers in this site probably do have some degree of water control and access to river water, which was not simulated, due to lack of data. It is unlikely that farmers would grow rice at this site without some degree of water control.

The box plots in Fig. 3 show that variation in drought risk within zones is often as large as variation in drought risk between zones. This comparison of drought risk between lowland and upland soils (short-scale variation) and between climate zones (large-scale variation) reveals that short-scale (topographic) variation is a greater determinant of drought risk than large-scale (climatic) variation is. This is something that is not noted in previous studies, which studied drought risk either only within individual valleys (short scale) or only between climate zones (large scale). Inland-valley catchments range in size from 100 to 2000 ha, depending on local topography. Within such catchments drought risk varies depending on landscape position along the hydromorphic zone (Danvi et al., 2016; Sakané et al., 2011; Schmitter et al., 2015; Windmeijer and Andriesse, 1993). Available climate zonations with higher resolution than the Köppen–Geiger (Van Wart et al., 2013) have minimum mapping unit sizes still many orders of magnitude larger than the size of inland valleys. Therefore, even using higher-resolution climate zone maps will not be able to capture this relevant short-range variation. The implication is that only high-resolution remote-sensing-based mapping, accounting for groundwater depth and duration of flooded conditions, will be able to identify drought hotspots and areas wet enough for rice cultivation. Mapping this is very labour demanding, requiring local collection of groundwater and flood data, and is unfeasible at a continental scale at the time of writing. Locally, drought characterisation can be relatively simple, a matter of asking farmers about frequency of flooding and depth of groundwater (Danvi et al., 2016; Sakané et al., 2011; Schmitter et al., 2015; Windmeijer and Andriesse, 1993).

**Fig. 4:**
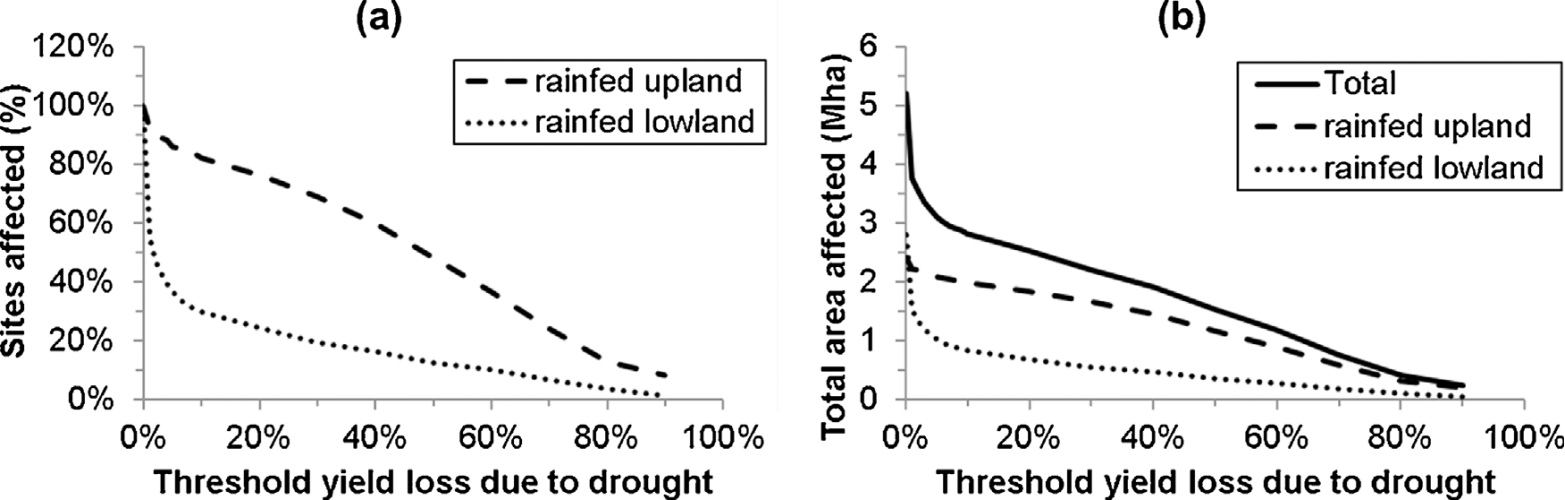
**Rice area in Africa potentially affected by drought as a function of threshold for severity of drought**: (a) percentage of sites and (b) total area (Mha). The threshold for severity of drought was calculated as 1–Yw/Yp, where Yw is the simulated maximum yield with drought (water limited) and Yp is the simulated maximum yield without drought (potential production).

Since the Köppen–Geiger climate zonation was not good for mapping drought stress, the potential rice area affected by drought was estimated from all site simulations taken together. At a 20% threshold yield loss (Yw/Yp < 0.8), 24% of all lowland sites and 76% of all upland sites were classified as suffering from drought (Fig. 4a). The lowland area potentially affected at this threshold is calculated as:

7.030 Mha (Table 1, SSA rice area) × 0.40 (Table 2, SSA fraction lowland) × 24% (Fig. 4a, lowland with Yw/Yp < 0.8) = 0.68 Mha.

The total potentially affected upland rice area was calculated similarly. Together, the total potentially drought affected area at a 20% threshold is 2.53 Mha (Fig. 4b), or 33% of Africa’s total rice area. At a higher threshold yield loss of 50% (Yw/Yp < 0.5), the area potentially affected is 1.520 Mha (20% of Africa’s total rice area).

### 3.3 Cold

**Fig. 5:**
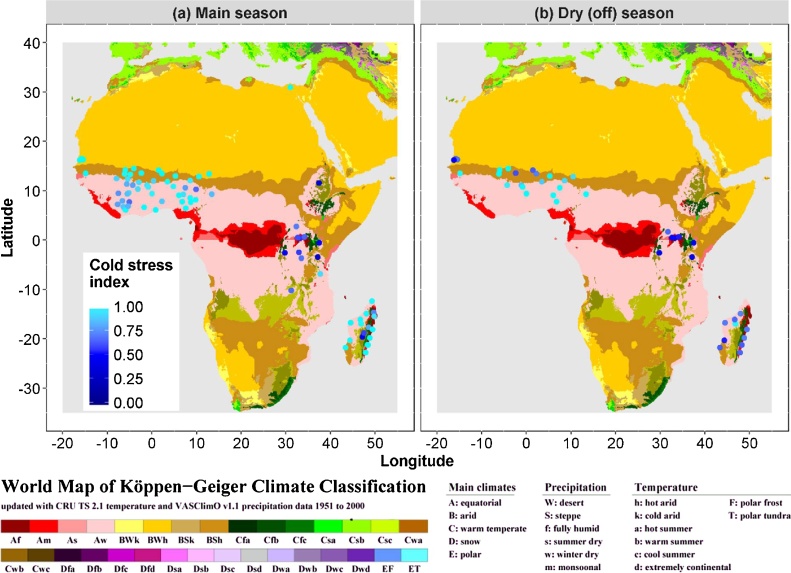
**Simulated cold stress**: (a) main season and (b) dry (off) season. Dots are sites for which cold stress was simulated using 5 years (irrigated rice) or 9 years (rainfed rice) of weather data for each dot. The cold stress index is SFCOLD, i.e. the cold-induced fertility: a low value means severe loss due to cold. Köppen–Geiger climate zones are shown in the background, legend and colour scheme from Kottek et al. (2006).

Fig. 5 presents the site-based cold stress simulations as blue dots, on top of the Koppen-Geiger climate zone map. The dot at 31°N, 31°E shows that cold stress does not occur in the Nile delta, where the critical flowering stage occurs during the sufficiently warm summer. Cold stress is consistently found (simulated) in the highlands of central East Africa (Uganda, Rwanda, north-east Tanzania, Kenya) and the Madagascar highlands, with severe cold sterility levels of 50–70%. For West Africa between 10°N and 17°N, approximately the Sahel zone, cold stress occurs in some of the irrigated systems in the dry season with cold sterility levels of 10–50% (SFCOLD 0.5–0.9). Closer analysis of these Sahelian cold risks revealed higher cold-sterility risk for earlier sowing dates (November, December) than for later sowing dates (January, February). Cold sterility was greater in coastal areas (Senegal) than inland (hotter areas of Mali, Niger, northern Benin and northern Nigeria). In Madagascar, in addition to cold stress in the highlands in the main (wet) season (50–70% sterility), ORYZA2000 predicted cold stress (30–50%) in the irrigated systems in the dry season along the whole coast (Fig. 5).

**Fig. 6:**
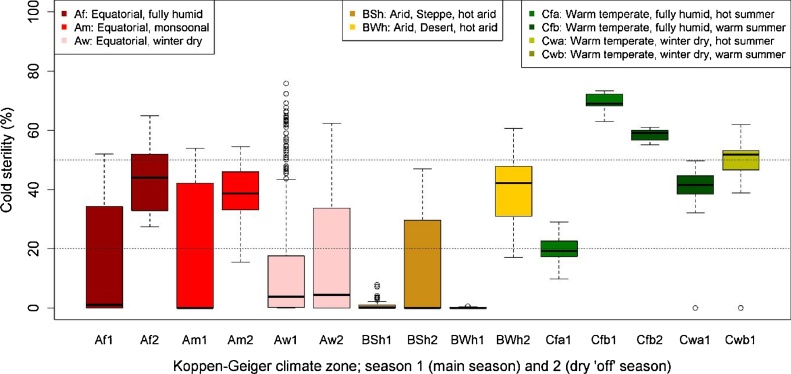
**Cold-induced sterility in the Köppen–Geiger climate zones: boxplots.** Cold sterility here is expressed as 100 × (1 − SFCOLD). Box plots are based on annual multi-site simulations, with 1 or 2 seasons. The black horizontal line in each box is the median. Top and bottom of the box are the 25th and 75th percentiles (Q1 and Q3). The “dots” at the end of boxplots represent outliers: Outliers are points with less than Q1 − (1.5 × IQR) or greater than Q3 + (1.5 × IQR), with IQR = Q3 − Q1. Box colours are consistent with the Köppen–Geiger colour scheme (Kottek et al., 2006).

For cold, contrary to drought, boxplots revealed that hotspots can be identified with the Köppen–Geiger climate zone map. The boxplots in Fig. 6 clearly show greatest cold risk in the temperate climates (Cfa, Cfb, Cwa, Cwb). In the equatorial climates (A), two zones (Af and Am) have some cold risk in the dry (“off”) season only, while one zone (Aw) has no cold risk. Irrigated rice in the desert climate zone (BWh) can experience cold in the dry (off) season, while in the steppe zone (BSh) limited cold risk is predicted. This results in the following simple classification:

1. Cold risk in all growing seasons: Cfa, Cfb, Cwa, Cwb
2. Cold risk in dry (off) season only: Af, Am, Bwh
3. No cold risk: Aw, BSh
4. Other: not quantified.

A conservative estimate of areas potentially affected by cold stress considers only the East African highlands, leading to an estimated potentially affected area of 0.261 Mha (Table 3), i.e. 3% of the total rice area of Africa. A more pessimistic projection includes the regions with moderate cold in the dry season and shows a potentially affected area of 0.570 Mha (7% of total rice area; Table 3).

**Table 3:** Estimated rice area potentially affected by cold.

Severe stress (sterility > 50%, SFCOLD < 50%)		Moderate stress (sterility > 20%, SFCOLD < 80%)	
Country/region potentially affected	Rice area potentially affected^a^ (Mha)	Country/region potentially affected	Rice area potentially affected^a^ (Mha)
Burundi	0.019	Burundi	0.019
Ethiopia	0.003	Ethiopia	0.003
Kenya	0.015	Kenya	0.015
Madagascar – only central highlands	0.167	Madagascar: central highlands + coastal zones in cold dry season	0.405
Malawi	0.041	Malawi	0.041
Rwanda	0.007	Rwanda	0.007
Tanzania – only Kia irrigated rice cold season	0.009	Tanzania – only Kia irrigated rice cold season	0.007
		Burkina Faso^b^	0.014
		Mauritania^b^	0.009
		Niger^b^	0.008
		Senegal^b^	0.042
Total (percentage of total rice area)	0.261 (3%)	Total (Mha)	0.570(7%)

a Average over the three crops maps.

b Only sites with irrigated rice in the cold dry season in the hot arid zones BSh and BWh.

### 3.4 Iron toxicity

**Fig. 7:**
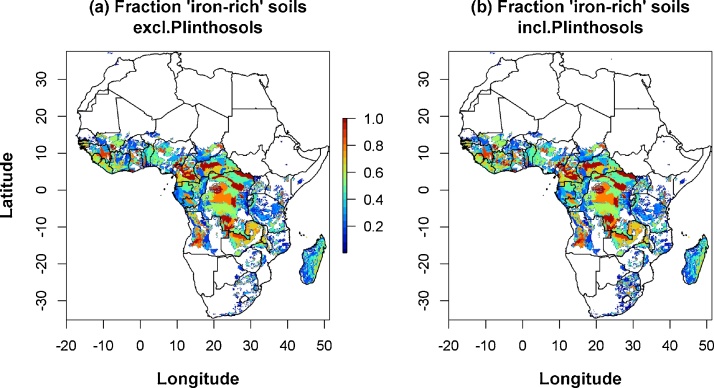
**Distribution of ‘iron-rich’ soils.** Based on the Harmonised World Soil Database (HWSD), see Materials and methods for selected soil types.

Fig. 7 shows the fraction “iron-rich soils” in Africa. Iron-rich soils are the dominant soil type in central Africa (Democratic Republic of Congo, central Angola, northern Zambia), but in these countries relatively little rice is grown. In West Africa, a number of hotspots are found (western part of Guinea, north-west Ghana) and in most places smaller fractions of “iron-rich soils” are found. In Madagascar, the whole east coast has a high fraction of “iron-rich soils”. Egypt has no “iron-rich soils”. The total estimated area with “iron-rich soils” in Africa is 427 Mha. As a result of the concentration of “iron-rich soils” in countries with relatively little rice (central Africa), only 0.897 Mha (0.2%) of the 427 Mha iron soils have rainfed lowland rice (Table 4, excluding Plinthosols).

**Table 4:** Estimated rice area potentially affected by iron toxicity.

	Excluding Plinthosols		Including Plinthosols	
	Area (Mha)	% of total rice area	Area (Mha)	% of total rice area
HWSD Africa total iron-rich area	427		440	
HWSD × SPAM2005 × RFL^a^	1.025	12%	1.066	12%
HWSD × MIRCA2000 × RFL	0.745	11%	0.799	12%
HWSD × GAEZv3 × RFL	0.921	12%	0.959	13%
Average rice on “iron-rich soils”	0.897	12%	1.066	12%

a Three rice crop maps (SPAM2005, MIRCA2000, GAEZv3) were overlaid with the Harmonised World Soil Database (HWSD) map of fraction “iron-rich soils” (Fig. 9) and then multiplied by country-specific fractions of rainfed lowland (RFL) from Diagne et al. (2013a).

Adding the Plinthosols made little difference to the total area of “iron-rich soils” (increase from 427 Mha to 440 Mha, +3%) and made no difference to the ranking of the main affected countries (data not shown). It did make a big difference to the total area of rice on “iron-rich soils” (0.897 Mha–1.066 Mha, +19%). The largest difference was found in Côte d’Ivoire, where the area potentially affected by iron toxicity increased from 0.055 Mha (14% of rice area) to 0.069 Mha (18%) when Plinthosols were included. A closer analysis of this case is presented in the discussion section of the paper and in the Appendix (§A.5).

**Fig. 8:**
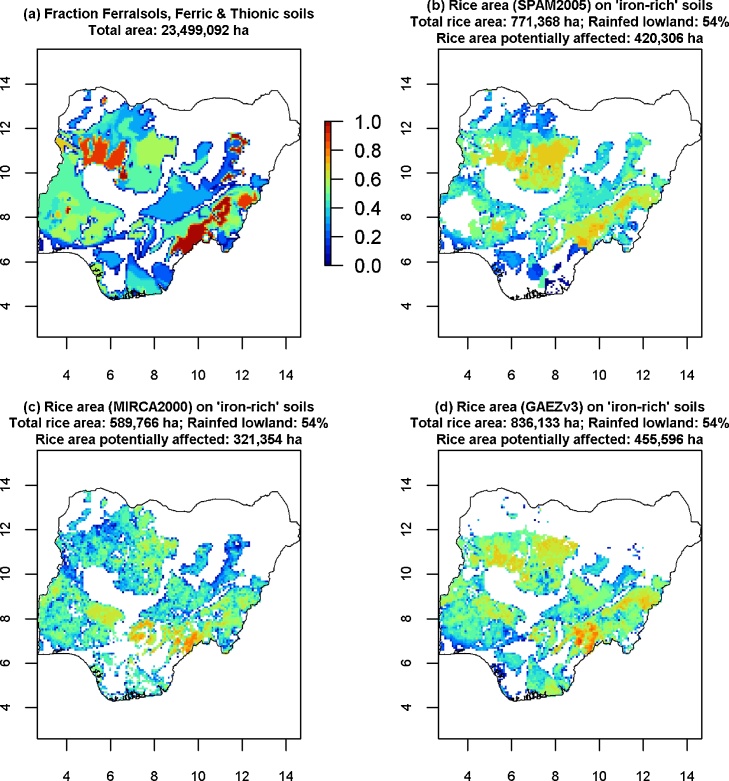
**Distribution of HWSD “iron-rich soils” in Nigeria and potentially affected rice area.** The top left pane shows the fraction of iron soils (same as in Fig. 7). The other figures show rice area potentially affected, with yellow/red indicating a larger area potentially affected. The fraction rainfed lowland (54%) is from Diagne et al. (2013a). For the three maps with potentially affected area, the legend has a logarithmic scale: log_10_(rice potentially affected area [ha]) per 9 × 9 km gridcell. Thus, a value of 4 (red) means 10^4^ = 10,000 ha, 3 (orange) means 10^3^ = 1000 ha, 10^2^ (green) = 100 ha and 10^1^ (blue) = 10 ha. Similar figures are available as downloads for all countries, in picture format and as kmz files for zooming in in Google Earth. (For interpretation of the references to colour in this figure legend, the reader is referred to the web version of this article.)

Table 5, Table 6 list the main countries in which rice is potentially affected by iron toxicity, using the classification without Plinthosols. Nigeria has the largest area potentially affected, while Togo, Ghana, Guinea-Bissau, Benin and The Gambia have the largest percentages of rice on “iron-rich soils” ([rainfed lowland rice area on “iron-rich soils”]/[total rice area]). This predominance of West African countries affected by iron toxicity follows from the greater concentration of “iron-rich soils” in West Africa compared with East Africa (Fig. 7). Fig. 8 shows as an example of the potentially affected rice areas in Nigeria, the main country affected. Fig. 8 and Table 5, Table 6, Table 7 show large uncertainty in estimated total area affected, which arises from using different crop maps. However, similar spatial patterns can be seen, which gives some confidence in the robustness of these maps for identifying hotspots.

**Table 5:** Top 10 countries with largest area potentially affected by iron toxicity.

Country	Rainfed lowland rice area (Mha) on “iron-rich soils” (excl. Plinthosols)			Percentage of rice on “iron-rich soils” ([rainfed lowland rice on iron]/[rice total])		
	SPAM2005	MIRCA2000	GAEZv3	SPAM2005	MIRCA2000	GAEZv3
Nigeria	0.42	0.32	0.46	17%	15%	21%
Guinea	0.12	0.09	0.13	16%	19%	20%
Madagascar	0.11	0.09	0.10	9%	9%	8%
Sierra Leone	0.08	0.02	0.02	12%	12%	12%
Tanzania	0.06	0.04	0.04	9%	12%	9%
Côte d’Ivoire	0.05	0.06	0.05	14%	13%	14%
Ghana	0.04	0.04	0.03	35%	32%	26%
Liberia	0.02	0.02	0.03	19%	15%	18%
Mali	0.02	0.01	0.02	5%	3%	6%
Guinea-Bissau	0.02	0.01	0.02	28%	26%	26%
Africa	1.03	0.75	0.92	12%	11%	12%
Average of crop maps		0.90			12%

**Table 6:** Top countries with largest percentage rice potentially affected by iron toxicity.

Country	Percentage of rice on “iron-rich soils” ([rainfed lowland rice on iron]/[rice total])		
	SPAM2005	MIRCA2000	GAEZv3
Togo	35%	28%	31%
Ghana	35%	32%	26%
Guinea-Bissau	28%	26%	26%
Benin	25%	23%	22%
The Gambia	21%	20%	15%

**Table 7:** Estimated rice area potentially affected by salinity/sodicity.

	Area (Mha)	Percentage of total rice area (∼7.7 Mha)
HWSD Africa total saline/sodic area	79	
HWSD × SPAM2005	0.149	2%
HWSD × MIRCA2000	0.169	2%
HWSD × GAEZv3	0.202	3%
Average rice on saline/sodic soils	0.173	2%

### 3.5 Salinity/sodicity

**Fig. 9:**
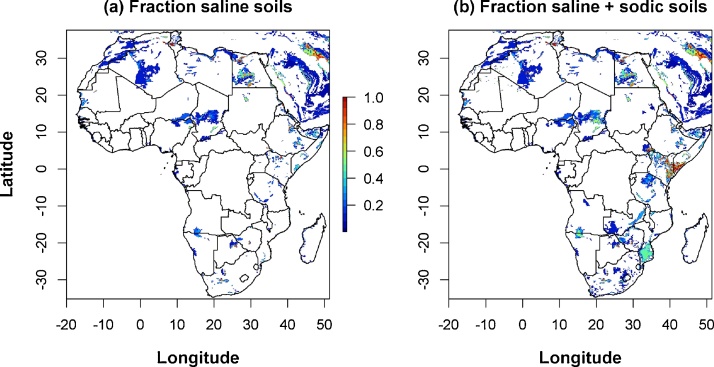
**Rice area with saline and saline/sodic soils.** Based on the Harmonised World Soil Database (HWSD), see Materials and methods for selected soil types.

Fig. 9 shows the fraction “saline” soils and “saline + sodic soils”. Saline/sodic soils are rarely the dominant soil type (red colour), except in northern and eastern Kenya and in the southern coastal plains of Somalia. Large inland areas with fractions of saline/sodic soil are found in the deserts of Algeria, Egypt and Chad, and in southern Mozambique. Rice is not an important crop in these areas, except along some river systems in Mozambique. Relatively large fractions of sodic soils are found in Mozambique, Kenya and Tanzania. The total area potentially affected by salinity/sodicity is 0.173 Mha (Table 7), or 2% of the total rice area of Africa.

No overlay with rice maps is given, as much of the rice on saline soils is in narrow coastal strips, which cannot be seen at the spatial and graphical resolution when shown at national or international scale. That large share of the salinity found in coastal areas, mainly in West Africa and along the west coast of Madagascar, is equally not visible on the map in Fig. 9, due to its spatial resolution. It can be seen clearly when zooming in on the maps in Google Earth, which are not presented here. To a certain extent it can also be seen from the list of the main affected countries (Table 8) and from countries with a relatively large mangrove rice area. Main countries with mangrove rice (Diagne et al., 2013a) also show as main countries with salinity (Nigeria, Guinea, Egypt, Sierra Leone, Guinea-Bissau). Tanzania stands out as a country without mangrove rice but with salinity/sodicity problems according to HWSD, which is attributed to inland sodic soils. According to Diagne et al. (2013a), the DRC also has a large area of mangrove rice (0.023 Mha, 6% of Africa’s mangrove area), but was not identified from HWSD and the crop maps as a country with major salinity problems.

**Table 8:** Top 10 countries potentially affected by salinity/sodicity.

Country	Rice area (Mha) on saline/sodic soils^a^			Percentage of rice on saline/sodic soils ([rice on saline]/[rice total])			Mangrove rice^b^	
	SPAM2005	MIRCA2000	GAEZv3	SPAM2005	MIRCA2000	GAEZv3	Mha	%
Egypt	0.03	0.08	0.08	4%	12%	13%	0.03	8%
Tanzania	0.04	0.02	0.03	6%	5%	6%	0.00	0%
Nigeria	0.02	0.03	0.01	1%	1%	0%	0.22	59%
Madagascar	0.02	0.02	0.02	1%	2%	2%	0.00	0%
Guinea	0.01	0.00	0.01	1%	0%	1%	0.04	11%
Senegal	0.01	0.01	0.01	7%	9%	15%	0.00	0%
Mozambique	0.01	0.01	0.03	7%	7%	16%	0.00	0%
Sierra Leone	0.01	0.00	0.00	1%	1%	1%	0.03	8%
Guinea-Bissau	0.00	0.00	0.00	8%	5%	4%	0.01	3%
Chad	0.00	0.00	0.00	4%	4%	5%	0.00	1%
Africa	0.149	0.169	0.202	2%	2%	3%	0.37	100%
Average of crop maps		0.173			2%			

a Based on the HWSD soil map, see Materials and methods for details.

b Based on Diagne et al. (2013a). These data serve as a reference but cannot be compared 1:1 with areas estimated based on the HWSD. See Materials and methods for details.

Egypt is the most affected country according to MIRCA2000 and GAEZv3; Tanzania is the top country according to SPAM2005. SPAM2005 shows a relatively smaller fraction of rice in the Egyptian Nile delta (and more rice to the south along the Nile) in comparison with MIRCA2000 and GAEZv3. This, in combination with concentration of salinity in the delta (HWSD), leads to a lower estimated area potentially affected according to the HWSD × SPAM2005 overlay.

### 3.6 Synthesis

Course-scale maps of four abiotic stresses for rice are presented. They do not capture the short-scale (high-resolution) spatial variability that also exists. Capturing this short-scale variability was not an objective of the current study, the objective of this study was to get an overall picture at a continental scale. This first course-scale analysis of where stresses are likely to be found can provide guidance for (for example) research prioritization and identifying target areas for seed distribution. For these objectives a course-scale map suffices.

#### 3.6.1 Total areas potentially affected

Table 9 shows total estimated areas potentially affected for the four stresses based on the previous sections. For reference, I add the two main stresses from the two other continent-wide studies. Table 9 suggests that low soil fertility (Haefele et al., 2014) and drought (this study) are the two main constraints (∼35% of Africa’s rice area), followed by weeds and iron toxicity (∼15%) and then cold (7%) and salinity/sodicity (2%). For weeds, the real area affected is probably larger than the 17% estimated based on Rodenburg et al. (2016), because that study considered only two weeds, which (although of great importance) do not cover the full spectrum of weeds troubling African rice farmers. The three stresses most important according to this paper – drought, fertility and weeds (Table 9) – are the same as previously reported by Waddington et al. (2010) as being the most important stresses for rice in Africa. This concurrence strengthens the confidence in the results. It should be noted that these area estimates are inevitably quite uncertain, due to uncertainties in the input data and assumptions, which are discussed in more detail in the following section.

**Table 9:** Summary of best estimates of total area potentially affected by four abiotic stresses in this study and prominent stresses quantified in previous studies.

Study	Stress	Rice area in Africa potentially affected (Mha, %)^a^	Description
This study	Drought	2.530 (33%)	Rainfed lowland and rainfed upland areas with >20% yield loss relative to potential (no drought stress) yield
This study	Cold	0.570 (7%)	Areas with >20% cold sterility. East African highlands + Sahel zone dry-season irrigated rice + Madagascar coast dry-season irrigated rice
This study	Iron toxicity	0.897 (12%)	“iron-rich soils” in rainfed lowland
This study	Salinity & sodicity	0.173 (2%)	Solonchaks + Salic Fluvisols + Solonetz
Rodenburg et al. (2016)	Parasitic weeds	1.34 (17%)^b^	*Striga asiatica*, *S. aspera*, *S. hermonthica* and *Rhamphicarpa fistulosa*
Haefele et al. (2014)	Low soil fertility	2.886 (37.6%)^c^	Topsoil >20% base saturation or cation-exchange capacity (CEC) > 20 cmol/kg clay

a The estimate which I personally deem most realistic, despite all uncertainties. In brackets is the estimated fraction of total rice harvested area in Africa (7.675 Mha, average of three crop maps).

b Rodenburg et al. (2016) do not report total rice harvested area in Africa. Here I assumed it is the same 7.675 Mha as in Table 1 and from this calculated the 17% affected area figure. This should give a reliable figure because Rodenburg et al. (2016) use a combination of MIRCA2000 and SPAM2005 as rice maps (average 7.7305 Mha).

c Total rice harvested area in Africa according to Haefele et al. (2014) is much larger (10.466 Mha), because they developed their own more recent rice-distribution map based on more recent FAOSTAT rice area data. Here, for consistency, I have kept the 37.6% given by Haefele et al. and thus calculated the area potentially affected as 0.376 × 7.675 = 2.886 Mha. See also the discussion on area expansion in Section 4.1.

#### 3.6.2 Hotspots

Hotspots could be identified for cold, iron toxicity and salinity/sodicity. For drought, the analysis shows that, with the course-scale maps used here, identifying hotspots is not really possible; for drought, it was shown that short-scale variation, reflecting topographic positions with different groundwater depths, is a greater determinant of drought risk than larger-scale climatic variation. Therefore, drought hotspots can only be determined via local high-resolution spatial analysis.

## 4 Discussion

To the best of my knowledge, this study is the first effort to map these four abiotic stresses for rice. Only drought was mapped once before, but in a manner not taking into account rainfall, groundwater and bunds (Haefele et al., 2014). There is a risk that in focusing on the three main stresses (drought, fertility, weeds), lesser stresses such as cold, salinity and iron toxicity remain unmapped. Here, for the first time, these three stresses were also mapped. For each stress, the main affected countries were identified and main uncertainties quantified. In the synthesis section (§3.6 above), I have sought to present the most accurate estimates despite these uncertainties. Below, I discuss the main uncertainties per stress in more detail, with reference to previous studies.

### 4.1 Crop area uncertainties

The most striking uncertainties in the three rice maps was in their allocation of rainfed and irrigated rice (§2.2.1, §A.1). The three crop maps are so uncertain in terms of their allocation of rainfed and irrigated rice that they were not used to differentiate these macro agro-ecosystems. Instead, maps of total rice area were used and these were multiplied by the country-specific fractions of irrigated rice, rainfed lowland rice, rainfed upland rice and “other” (mainly mangrove rice) from Diagne et al. (2013a). Therefore, we still do not know the spatial distribution of these growing environments within the countries. This is especially an issue for those stresses that are strongly tied to specific growing environments: drought in rainfed upland, iron toxicity in poorly drained lowlands, and salinity in coastal mangrove areas.

**Fig. 10:**
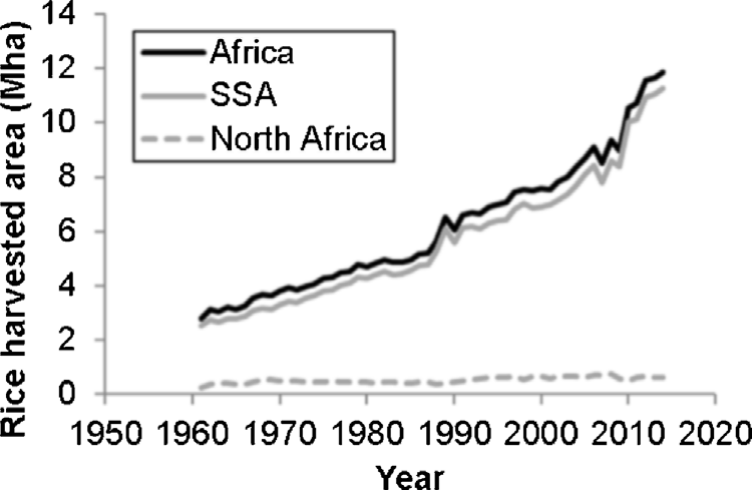
Expansion of rice area in Africa.

Source: FAOSTAT.

A second uncertainty in the crop maps used in this study is that they contain estimated crop areas from around 2000 (MIRCA2000, GAEZv3) and 2005 (SPAM2005). According to FAOSTAT (Fig. 10), from 2000 to 2014 rice area increased from 7.6 Mha to 11.9 Mha, a 57% increase that came from area expansion in sub-Sahara Africa (SSA, 6.9 Mha–11.3 Mha, +64%), while in North Africa, rice area decreased slightly (0.671 Mha–0.594 Mha). If expansion took place into more marginal environments, which seems likely since the best lands are often brought into cultivation first (Young, 1999), then both in relative and in absolute terms stresses will be bigger now than they were in 2000–2005. I reflect on this in more detail below (§4.6).

### 4.2 Drought uncertainties

The drought analyses considered the most important determinants of drought stress in rice (rainfall, groundwater depth, percolation rates, sowing dates). Previous sensitivity analyses showed that for rice, with its shallow rooting system, groundwater-table depth, bunding and percolation rates are far more important variables than soil water-retention properties (Boling et al., 2007; Bouman et al., 1994, Bouman et al., 2007; Wopereis et al., 1994). Also, the current study showed how strongly dependent rice yields are on groundwater depth. For future research on rainfed rice, the lack of high-resolution groundwater-depth datasets represents a major constraint.

A conceptual critique on the drought-stress analysis presented in this paper is that it may show bias in particular cases:

1. For upland rice, the comparison of water-limited yield (Yw) with drought-stress free potential yield (Yp) may seem hypothetical, because Yp could only be achieved with irrigation which may simply not be an option if there are no large water bodies available in an area. One might argue the benchmark Yp is therefore too high. Following this rationale, one could argue that this study overestimates the area potentially affected by drought.
2. Drought stress was simulated for all locations for rainfed upland and rainfed lowland rice. Most likely farmers would not be growing upland rice if the risk of drought was too high, in which case this study could be overestimating the area potentially affected by drought.
3. The analysis assumed no drought in irrigated rice. But, drought can also occur in irrigated systems, especially at the tail ends of irrigation schemes or as a result of poor water management or pump failure. Consequently, the current study may have underestimated drought.

Some of these errors of overestimation and underestimation may cancel each other out when calculating the overall area potentially affected. Despite all this uncertainty, however, some general patterns emerged. Firstly, even with more conservative drought-stress estimates, drought still ranks as the number one stress, much more important than the second ranked stress (iron toxicity, 12% of rice area). Secondly, the analysis revealed that short-scale variation in drought risk along the hydromorphic zone is much more important than large-scale variation due to climate. This means that climate zone maps are of limited use for identifying drought hotspots, and rather that higher-resolution mapping is required for identifying drought hotspots.

Reality is, of course, far more complex than the “simplified” modelling of varieties with similar cycles for upland and lowland. However, for each location the temperature sums were calibrated so that crop duration (sowing to maturity) matched local cropping calendars. Closer analysis of this dataset shows that crop duration in this dataset is significantly shorter in drier regions. This is what one would logically expect as a drought-avoidance strategy of farmers. With a shorter-cycle cultivar, the impact of drought stress could be different. In general, potential yield (Yp) is lower for shorter-duration varieties, because they have less time to accumulate biomass. Water-limited yield (Yw) would be expected to remain similar or even slightly higher (if reducing terminal drought) or Yw could decrease (in case of mid-season drought, which would occur regardless of crop duration). In these two cases, the drought indicator Yw/Yp would increase (less stress) or remain similar (similar stress). This would be very interesting to analyse in more detail with better datasets on groundwater depth and varieties, but such were not available at the time of writing.

### 4.3 Cold uncertainty

As discussed in Section 2.5, the scientific basis for predicting cold stress in rice across Africa is still thin and requires further research. A physiologically sufficiently sophisticated model would not require applying different parameters for the same variety in different regions such as I did in this study. Further empirical and modelling work is needed in this regard. The equations used here were those calibrated by Dingkuhn et al. (2015) for variety IR64, which performs well in tropical environments. If cold is indeed persistent in the East African highlands with their temperate climate, then it seems likely that farmers would have adapted by choosing varieties more cold-tolerant than IR64. In that case, the current study may have been overestimating cold stress in this part of Africa. This study did not differentiate cold-stress risk at high resolution along altitudinal zones in the East African highlands. Such high-resolution mapping would be highly relevant, but was impossible at the time of writing due to lack of high-spatial-resolution weather data and remaining uncertainties in cold-sterility models.

van Oort et al. (2015a) report that at moderate sterility good yields can still be obtained if the number of spikelets is high and if there is ample growth during the grain-filling phase. For example, if a rice crop produces 40,000 spikelets per m^2^ (which is not unrealistic in fertile environments, e.g. see de Vries et al., 2011), then with a grain weight of 25 mg and a spikelet fertility of 70%, potential yield is 7.0 t/ha, thus still quite high despite 30% sterility. With low soil fertility levels in Africa (Haefele et al., 2014) generally leading to low spikelet numbers (spikelets/m^2^) and slower grain-filling, moderate levels of cold sterility of 20% can be detrimental. Considering two threshold levels for cold sterility, the estimated area potentially affected was estimated at 0.26–0.57 Mha, or 3–7% of total rice area. Despite all these uncertainties, some general patterns emerged that could be linked to the Köppen–Geiger climate zonation. Cold is consistent in the highlands of East Africa, and is a problem for rice in West Africa in the Sahel zone (if sowing early in the cold dry season) and in coastal regions of Madagascar (also in the cold dry season).

### 4.4 Iron-toxicity uncertainty

As with drought, iron toxicity can vary strongly at short spatial scale, caused by variations in local topography and the presence of drainage, something which could not be mapped in the current analysis. The rationale for iron-toxicity mapping in this study was simply that iron toxicity is more likely on “iron-rich soils”, for which only a course-resolution soil map was available at continental level. Since no high-resolution map of rainfed lowland rice was available, a 9 × 9 km map of total rice area was multiplied with the national fraction of rainfed lowland, so spatial precision is limited for this stress.

To the best of my knowledge, this is the first effort ever to map the risk of iron toxicity at the scale of Africa. Validation is therefore not possible at the continental scale. The only previous published regional mapping effort for iron toxicity in Africa is Chérif et al. (2009). That study measured iron toxicity by visually assessing leaf bronzing. A cross-tabulation for Côte d’Ivoire (Table 10, see also the Appendix §A.5) revealed:

• six out of the nine sites show a positive correlation between leaf-bronzing score and share of “iron-rich soils”, i.e. low bronzing scores on soils with a low share of “iron-rich soils” and medium bronzing scores on soils with a medium share of “iron-rich soils”;
• four sites visited by Chérif et al. (2009) are on boundaries between HWSD units, which could therefore not be used for validation;
• None of the parts of the country with high iron-toxicity risk according to HWSD were visited by Chérif et al. (2009);
• lower risk of leaf bronzing at two sites with plinthite soils (see the Appendix §A.3).

**Table 10:** Validation of iron-toxicity risk in Côte d’Ivoire.^a^

Visual scoring of bronzing of leaves^b^, ^c^	Fraction HWSD “iron-rich soils”			Sampling sites on boundary between HWSD units with medium/low iron content^b^
	Low (<30%)	Medium (30–70%)	High^c^ (>70%)	
Low	Abengourou	Daloa		Bondoukou
(1,3)	Boundiali	Odienné		Dabakala
	Soubré			
	Touba			
Medium	Bouna	Gagnoa		Danané
(5,7)		Korhogo		Man

a See Appendix (§A.5) for background information on the validation.

b Chérif et al. (2009).

c None of the parts of the country with high iron-toxicity risk according to the HWSD soil map were visited by Chérif et al. (2009).

This validation also shows the complexity of making comparisons between two datasets based on different approaches at different spatial scales. Overall, the image of iron toxicity that emerged during the literature review for the current study was that most research on iron toxicity has been on rice physiology. While some soil types are identified in the literature as having a high risk of iron toxicity, it remains unclear from the literature whether all of these “iron-rich soils” have equal risk or whether some have higher risk than others. By considering all of them equally risky, the current study may have overestimated the area potentially affected by iron toxicity. While hotspots could be identified in the current study, I consider that the maps developed here are only a starting point for further research. This research with course-scale mapping of iron-toxicity risk needs to be followed up with higher-spatial-resolution studies.

### 4.5 Salinity/sodicity uncertainty

Cross-checking salinity maps with estimates of mangrove areas showed some consistency, but also identified countries with sodic soils but no mangrove rice (see §3.5). This suggests that mapping mangrove rice alone does not give the full picture, because it fails to map inland areas with salinity/sodicity caused by saline parent soil material. With saline/mangrove areas often concentrated in a relatively narrow strip along the coast, area estimates based on crop maps overlaid with HWSD are sensitive to the accuracy of the crop maps, particularly along the coastlines.

Cross-checking the top salinity/sodicity countries with the available literature on salinity in rice in Africa yields a mixed picture (Table 11). In many cases, studies on salinity/sodicity in rice were conducted in areas which are also saline/sodic according to HWSD, which confirms the soundness of the approach developed here. In Nigeria there was a mismatch: salinity according to HWSD occurs in other parts of Nigeria than where research has been conducted on salinity. In some sites in Madagascar, Chad and north-west Tanzania (Mwanza, Shinyanga), HWSD predicted salinity/sodicity and the presence of rice, while there are no publications on rice and salinity from those sites. Of course, the lack of publications from particular countries does not mean these countries have no salinity/sodicity problems in rice. There are several other reasons why there may be no publications, for example grey literature may exist in French and researchers might never have come to writing English studies; this was difficult to find out. The maps presented here could be valuable for identifying new hotspots in regions where the salinity problem might possibly not yet be signalled by research agencies. They can also be relevant when large rice area expansion is planned and suitable target areas are sought. It remains to be tested whether the maps are indeed useful or whether they are simply wrong (i.e. that there is no salinity problem after all in certain “hotspots”).

**Table 11:** Literature on salinity/sodicity in 10 worst affected countries according to this study.

Country (Table 8)	Reference
Tanzania	Kashenge-Killenga et al. (2016): South-west, both salinity and sodicity, consistent with HWSD. No study found on salinity or sodicity in the north-west (Mwanza, Shinyanga)
Egypt	Delta: (El-Shahway et al., 2016; Kotb et al., 2000) consistent with HWSD
Nigeria	Two studies in areas with no salinity according to HWSD: (Akinbile et al., 2016; Akpan et al., 2015). No studies in areas with high salinity problems for rice according to HWSD and crop maps.
Madagascar	No studies on salinity in rice in Madagascar.
Guinea	Coastal zone (Sylla et al., 1995; Wolanski and Cassagne, 2000) north of Conakry. Consistent with HWSD, but HWSD shows low share of saline soils.
Mauritania	No salinity/sodicity in Foum Gleita, middle part of the Senegal River valley (van Asten et al., 2003b) – consistent with HWSD.
Senegal	Salinity in Casamance (Sylla et al., 1995; Thiam and Singh, 1998), consistent with HWSD. Salinity in about 10% of the planned irrigation scheme along N’Galenka creek, middle part of the Senegal River valley (Barbiero et al., 2001) – no salinity there according to HWSD, which might be consistent with such a relatively small fraction of saline soils.
Mozambique	Menete et al. (2008): South of Mozambique, consistent with HWSD.
Sierra Leone	Coastal zone (Sylla et al., 1995), consistent with HWSD.
Guinea-Bissau	Coastal zone (Sylla et al., 1995), consistent with HWSD.
Chad	No studies on salinity in rice in Chad.

The analysis showed that salinity is found both in coastal regions and inland. Mapping salinity risk in the coastal regions remains a challenge. During the wet season or with irrigation, fresh rain, river and/or irrigation water “pushes down” and dilutes salts in the upper soil layer. The degree to which this happens depends strongly on the amount of fresh water and its salinity (river water can also be saline). Salinity can therefore have strong seasonal dynamics (Wolanski and Cassagne, 2000). While HWSD correctly shows salinity in many coastal areas, it does not capture this seasonality. Another source of uncertainty is dams. Construction of dams near coastal regions can prevent sea-water intrusion upstream from the dam and it can allow for more controlled irrigation water supply upstream from the dam, where salinity could be reduced with ample irrigation and drainage. A drawback can be that, if less fresh water flows through the dam towards the sea then more sea water can intrude in the downstream area from the sea up to the dam, leading to increased salinity in this area. Apart from lacking data on dams, data were lacking on the magnitude of these phenomena. A third source of uncertainty on salinity is about the extent to which drainage systems are in place and functioning properly in irrigated systems and the extent to which farmers re-use increasingly saline drainage water (El-Shahway et al., 2016; Kotb et al., 2000). Uncertainty on coastal water dynamics, dams and drainage water use causes considerable uncertainty about the estimate of the area potentially affected by salinity.

### 4.6 Future outlook

For the future, further large crop area expansion is expected in Africa for rice and other cereals (van Ittersum et al., 2016; van Oort et al., 2015b). Often the best soils are already in use (Young, 1999), therefore one might expect expansion into areas with increasingly poor soils. Since these are currently not yet cultivated, or only marginally so, their properties are often also unknown, because agricultural research and development tends to focus on existing crop areas. Course-scale maps such as those presented here can provide a first cue for what to watch out for. If expansion takes place in wetlands, then iron-toxicity, flooding and typical wetland weeds (e.g. *Rhamphicarpa fistulosa*; Rodenburg et al., 2016) will likely increase. In East Africa, cold risk could increase if rice area expands in the highlands. In West Africa in the desert zone and in Madagascar coastal zones, cold risk could increase if double rice cropping in irrigated systems (van Oort et al., 2016) becomes more prevalent. Fertilizer application can to some extent can mitigate the problems of salinity and iron toxicity (van Asten et al., 2003a, van Asten et al., 2003b, van Asten et al., 2004; Sahrawat, 2004) and cold (increasing number of spikelets and growth during grain-filling): if fertiliser applications are increased, then the severity of these stresses might decrease. On a longer timescale, cold sterility can be expected to decrease due to climate change (Van Oort and Zwart, 2017). The pumping revolution, along with solar panels becoming cheaper and cheaper, is also slowly taking off in Africa (Pavelic et al., 2013; Villholth, 2013). If this trend continues, it could lead to reduced drought risks, but possibly more conflicts over river and groundwater use. It could also lead to increasing salinity, if the pump revolution is not accompanied by development of drainage networks. The stress mapping reported here is therefore a snapshot of stresses around the time of writing and may require updating in the future.

## 5 Conclusions

This study provides maps and area estimates of four abiotic stresses for rice in Africa. In terms of relative importance, the study identified drought as the most important stress (33% of rice area potentially affected), followed by iron toxicity (12%), then cold (7%) and salinity/sodicity (2%). The course-scale maps developed in this study can be used to send stress-tolerant varieties to the stress hotspots identified. Hotspots could be identified for iron toxicity, cold and salinity/sodicity. Drought appeared to occur everywhere and could not be well mapped using the climate zonation used in this study, because short-scale spatial variation along hydromorphic zones is more important than large-scale climate variation. Since high-resolution (in space and time) groundwater maps are not available, drought could not be mapped well. Still, a sensitivity analysis showed that, despite this, drought remains by far the most important stress for rice among the stresses investigated here.

## Downloads

All supplementary maps and tables can be downloaded from https://dataverse.harvard.edu/dataverse/AfricaRice?q=Abiotic+stress+maps+for+rice+(STRASA). This web page contains maps of the four abiotic stresses (drought, cold, iron toxicity and salinity/sodicity) for rice in Africa, tiff files and tables for documents and presentations, and kml/kmz files for visualization in Google Earth.

## Figures and Tables

**Fig. A1:**
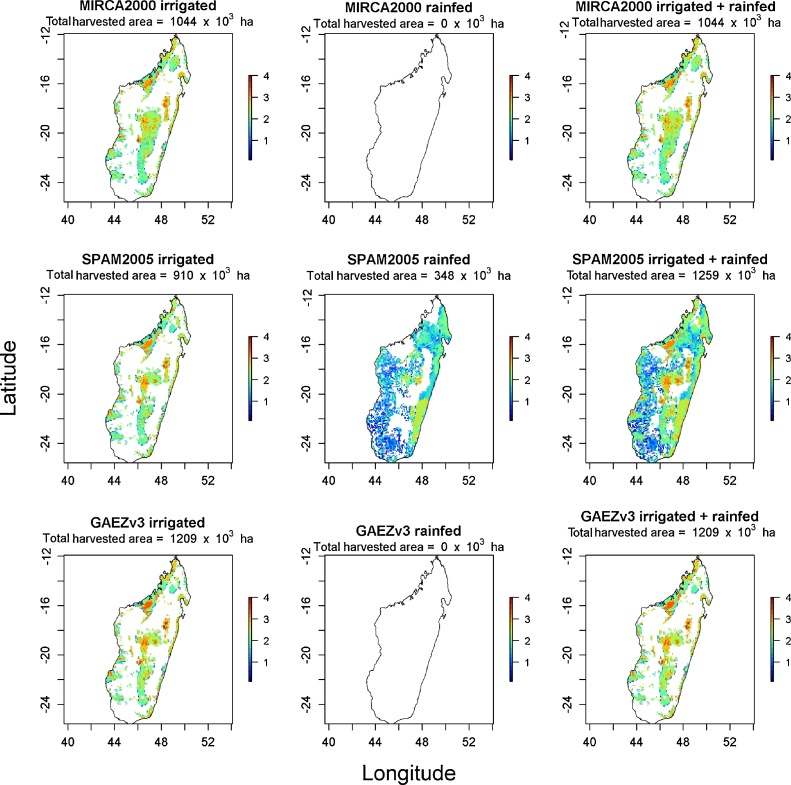
**Madagascar rice areas according to the three crop maps.** The legend has a logarithmic scale: log_10_(rice harvested area [ha]) per 9 × 9 km gridcell. Thus, a value of 4 (red) means 10^4^ = 10,000 ha, 3 means 10^3^ = 1000 ha. Note: according to MIRCA2000 and GAEZv3, there is no rainfed rice in Madagascar, which is unrealistic. (For interpretation of the references to colour in this figure legend, the reader is referred to the web version of this article.)

**Fig. A2:**
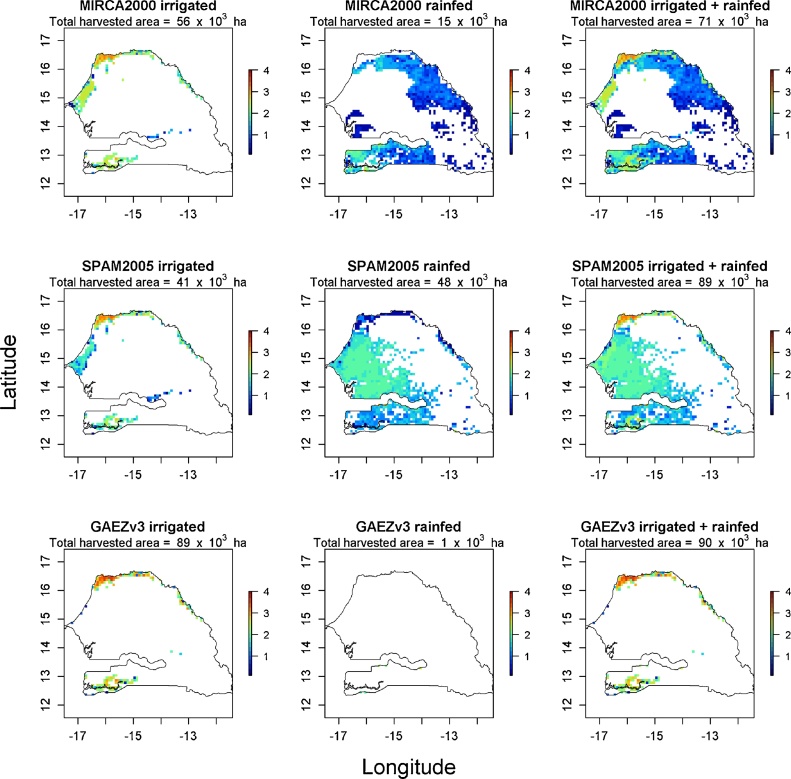
**Senegal rice areas according to the three crop maps.** The legend has a logarithmic scale: log_10_(rice harvested area [ha]) per 9 × 9 km gridcell. Thus, a value of 4 (red) means 10^4^ = 10,000 ha, 3 means 10^3^ = 1000 ha, etc. Note: according to SPAM2005, there is a substantial area of rainfed rice in the zone 13.5–16°N, which is unrealistic because this region is too dry for growing rainfed rice. (For interpretation of the references to colour in this figure legend, the reader is referred to the web version of this article.)

**Table A1:** Soil parameters.

Parameter	Lowland^a^	Upland^b^
Soil texture	Clayey	Sandy
Saturated moisture content *θ_s_* (WCST) (m^3^/m^3^)^c^	0.57	0.39
Van Genuchten parameter α (VGA) (/cm)^c^	0.0205	0.0321
Van Genuchten parameter *l* (VGL)^c^	40.7	60.9
Van Genuchten parameter *n* (VGN)^c^	1.2848	2.163
Van Genuchten parameter *θ_r_* (VGR)^c^	0.27	0.04
Bund height (mm)^d^	250	0
Groundwater depth (cm)	40	1000
Saturated hydraulic conductivity KSAT (cm/day)	10.789	99.77
Percolation rate (mm/day)^e^	3.7	240.3
Puddling^f^	No	No
Initial moisture content: Field capacity	0.562	0.375

a EC4-fine (van Diepen et al., 1989).

b EC1-course (van Diepen et al., 1989).

c van Genuchten (1980) equations: soil moisture content *θ:*$\theta =\theta_{r}+\left(\theta_{s}-\theta_{r}\right){\left[1+{\left|\alpha \right|}^{n}\right]}^{-m}$ with *m* = 1 − 1/*n*.

d Bund height 0 for upland soils means there are no bunds, which is the common situation for most upland rice in Africa.

e I chose the option in ORYZA2000 to simulate with a fixed percolation in cases of standing (aboveground) water. The high upland soil percolation rate implies that any standing would immediately drain. Without standing water, downward flow is calculated based on hydraulic conductivity calculated with the van Genuchten equation (Bouman et al., 2001), which is less than the percolation.

f Few farmers in Africa practise puddling, even in irrigated systems.

**Table A2:** Potentially iron-toxic soils, with and without plinthic properties.

	Ferralsol, Ferric and Thionic	
	Excluding Plinthosols	Including Plinthosols
Soil units with FAO-74 classification	F Ferralsols	+
	Fo Orthic Ferralsols	Ap Plinthic Acrisols
	Fx Xantic Ferralsols	Fop Plinthic Ferralsols
	Fr Rhodic Ferralsols	Gp Plinthic Gleysols
	Fahd Humic Ferralsols	Lap Plinthic Luvisols
	Far Acrid Ferralsols	LXp Plinthic Lixisols
	Af Ferric Acrisols	
	Lf Ferric Luvisols	
	Pf Ferric Podzols	
	Jt Thionic Fluvisols	

Soil units with FAO-90 classification	FR Ferralsols	+
	FRh Haplic Ferralsols	PT Plinthosols
	FRx Xanthic Ferralsols	PTe Eutric Plinthosols
	FRr Rhodic Ferralsols	PTd Dystric Plinthosols
	FRu Humic Ferralsols	PTu Humic Plinthosols
	FRg Geric Ferralsols	PTa Albic Plinthosols
	FRp Plinthic Ferralsols	ACp Plinthic Acrisols
	LVf Ferric Luvisols	ALp Plinthic Alisols
	LXf Ferric Lixisols	LXp Plinthic Lixisols
	ACf Ferric Acrisols	
	ALf Ferric Alisols	
	PZf Ferric Podzols	
	FLt Thionic Fluvisols	
	GLt Thionic Gleysols	
	HSt Thionic Histosols	

**Fig. A3:**
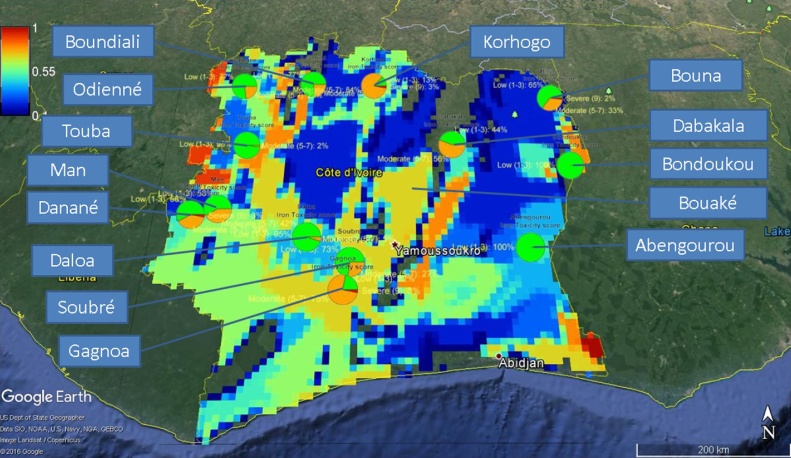
**Share of “iron-rich soils” (excluding Plinthosols) in Côte d’Ivoire in Google Earth.** The pie charts show iron-toxicity scores observed from leaf colouring from a validation dataset (Chérif et al., 2009). The grid shows calculated fraction of “iron-rich soils” based on HWSD. Similar maps for visualization in Google Earth are available for all African countries.

**Fig. A4:**
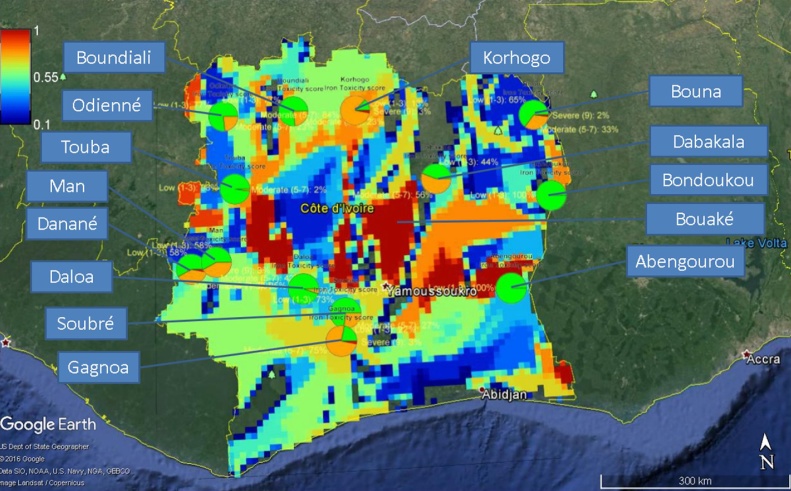
**Share of “iron-rich soils” (including Plinthosols) in Côte d’Ivoire in Google Earth.** The pie charts show iron-toxicity scores observed from leaf colouring in a validation dataset (Chérif et al., 2009). The grid shows calculated fraction of “iron-rich soils” based on HWSD. Similar maps for visualization in Google Earth are available for all African countries. Compared with Fig. A3, the main difference is a belt of Plinthosols half way south to north, running from the west around Touba to the east around Abengourou.

**Table A3:** Validation of iron-toxicity risk in Côte d’Ivoire.

Visual scoring of bronzing of leaves^a^	Fraction HWSD “iron-rich soils”			Sampling sites on boundary between HWSD units with medium/low iron content
	Low (<30%)	Medium (30–70%)	High^b^ (>70%)	
Low	Abengourou	Daloa		Bondoukou
(1,3)	Boundiali	Odienné		Dabakala
	Soubré			
	Touba			
Medium	Bouna	Gagnoa		Danané
(5,7)		Korhogo		Man

a Chérif et al. (2009).

b None of the parts of the country with high iron-toxicity risk according to the HWSD soil map were visited by Chérif et al. (2009).

## Appendix A A.1 Uncertainty in crop maps

### A.1 Uncertainty in crop maps

This part of the appendix shows the uncertainty in the three crop maps (SPAM2005, MIRCA2000 and GAEZv3) in terms of their spatial allocation of irrigated and rainfed rice. Two country examples are presented: Madagascar and Senegal. Madagascar has 0% rainfed rice area according to MIRCA2000 and GAEZv3 and 28% according to SPAM2005 (Fig. A1). According to survey data by Diagne et al. (2013a), which are in this case more realistic, rainfed rice area in Madagascar is 34% of the total rice area of Madagascar.

All three maps tend to allocate substantial rainfed rice areas in semi-arid zones, which is highly unlikely. This is illustrated for Senegal (Fig. A2). In Senegal, all three datasets correctly and consistently position the highest concentration of the rice-producing area along the Senegal River along the northern border with Mauritania (around 16°N, 16°W) and a second concentration of rice in the Casamance region (12.5–13°N). In Senegal, according to SPAM2005 there is a vast area of rainfed rice in the peanut belt (13.5–16°N). This part of Senegal is very dry (annual precipitation divided by mean annual potential evapotranspiration is less than 0.27), so it is impossible to grow rice there. In this case, SPAM2005, which was most realistic in Madagascar, is least realistic in Senegal. Differences in accuracy between the crop maps and between countries justify using all three for a sensitivity analysis, thus quantifying the uncertainty in stress potentially affected rice area arising from uncertainty in crop maps. However, there is no need to use obviously erroneous data. Therefore, I used only the maps of total rice area and not the available maps of rainfed and irrigated rice.

### A.2 Soil parameters for drought simulations

Table A1 lists the soil parameters for the two soil types considered in the current paper, representing the continuum from freely draining unbunded “typical” upland soils to “typical” lowland soils with bunds and shallow groundwater.

### A.3 Iron-rich soil units in the HWSD soil map

Iron toxicity was mapped with the Harmonised World Soil Database (HWSD). The HWSD (FAO et al., 2012). It has 16,327 unique map units. Within each map unit, there are non-georeferenced soil units or associations of soil units. Each soil unit has an FAO soil unit classification: SU_SYM74 according to the FAO-74 soil classification, SU_SYM85 according to the FAO-85 interim soil classification, and SU_SYM90 according to the FAO-90 soil classification. SU_SYM74 and SU_SYM90 together provide classifications for all soil units (i.e. each soil unit has a value for either SU_SYM74 or SU_SYM90). The HWSD is available as a raster dataset and as a polygon dataset. Here, I used the 0.0083° (approximately 0.9 × 0.9 km) resolution raster dataset. For each soil mapping unit (SMU), I calculated the fraction iron-rich soil units (Table A2) based on their share within each SMU. The share of iron was aggregated (spatial average) to the same spatial resolution of the three crop maps (∼9 × 9 km) and overlaid with the rice maps and multiplied by the national fraction of lowland rice (Diagne et al., 2013a) to calculate potentially affected rice area.

### 1 A.4 Salinity and sodicity in the HWSD soil map

The HWSD contains three relevant fields for identifying salinity and sodicity: the soil unit (SU_SYM74 or SU_SYM90), topsoil (0–30 cm) electrical conductivity (T_ECE), and topsoil (0–30 cm) exchangeable sodium percentage (T_ESP). I focus here on the topsoil since this is where most rice roots are. I calculated the share of Solonchaks + Salic Fluvisols (SCFLs, saline), share of Solonetz (SN, sodic), the share of Solonchaks + Salic Fluvisols (saline) + Solonetz (SCFLsSN, saline or sodic), the share of soil units with T_ECE > 4 dS/m (saline) and the share of soil units with T_ESP > 6% (sodic). The threshold values for T_ECE > 4 dS/m are consistent with thresholds reported for rice (FAO et al., 2012; Grattan et al., 2002; Tanji and Kielen, 2002), although varietal differences exist, with some varieties being more tolerant and some being less tolerant. According to FAO et al. (2012), ESP values in the range 6–15% cause moderate problems for most crops. This is much lower than threshold values reported by Gupta and Sharma (1990), which show that rice is, compared with other crops, quite tolerant of sodicity, with no yield losses at ESP values less than 24.4%. I therefore also considered this higher value, which for simplicity I set to 25%. An investigation of correlations between these shares, for the full HWSD dataset with its 16,327 unique soil map units, revealed the following relevant relations:

1. All saline soils are also sodic (99% of SCFLs with T_ESP > 6%)
2. Most sodic soils are **not** saline (98% of SN have T_ECE < 4 dS/m)
3. Around 71% of the Solonchaks + Salic Fluvisols have T_ECE values above 4 dS/m
4. Around 63% of the Solonchaks + Salic Fluvisols have T_ESP values above 25%
5. Around 98% of the Solonetz have T_ESP values above 6%
6. Around 2% of the Solonetz have T_ESP values above 25%.

It seems strange that many Solonchaks + Salic Fluvisols (29%), known for their salinity, have T_ECE values of less than 4 dS/m. Severe sodicity (T_ESP > 25%) occurs more in the Solonchaks + Salic Fluvisols (63%) than in the Solonetz (2%), which seems strange as Solonetz are much more known for their sodicity than Solonchaks are. Therefore, I decided to use the soil unit names and not their T_ECE and T_ESP values to identify saline and sodic soils.

### • A.5 Iron-toxicity validation

The only published regional effort for mapping iron toxicity in Africa was by Chérif et al. (2009) and did not include a soil survey. Chérif et al. (2009) collected a large georeferenced dataset on iron toxicity from different places in Côte d’Ivoire, Ghana and Guinea. I considered only the sample sizes from Côte d’Ivoire sufficiently large for a validation with the HWSD map of “iron-rich soils”. For 13 sites in Côte d’Ivoire, on average 211 observations per site were available, from different farmers and different lowlands in the vicinity of each site. From these, I drew pie charts showing the fraction of observations with low leaf-bronzing scores (1–3, green), medium leaf-bronzing scores (5–7, orange) and high leaf-bronzing scores (9, red). The two variables compared are not the same: I compare visual leaf-bronzing scores with share of “iron-rich soils” within HWSD soil mapping units (this study). Leaf-bronzing scores are often, but not always, correlated with iron toxicity. According to Sikirou et al. (2015), occasionally growth reduction and significant yield reduction can be observed in rice plants exposed to excess Fe^2+^ without leaf bronzing. This adds an element of uncertainty to the validation. Fig. A3 shows the Google Earth screenshot of pie charts on top of a map of share of “iron-rich soils” for the iron map without the Plinthosols. Fig. A4 shows the same with the Plinthosols included. Comparing the maps of Fig. A3, Fig. A4, we can see a belt of Plinthosols running from east to west, with Bouaké and Abengourou in this belt. According to the farm surveys, no iron toxicity was observed at Abengourou. Around Bouaké, close to the Africa Rice Centre research station, no surveys were conducted by Chérif et al. (2009), but I know from experimentation in this area that iron toxicity is not a major issue in this part of the country. Both sites suggest no iron-toxicity risk on Plinthosols, which is consistent with the lack of mention of Plinthosols in the iron-toxicity literature, but far from sufficient for drawing general conclusions on iron-toxicity risk on soils with plinthite. The validation based on Fig. A3 yields a mixed picture (Table A3), with no samplings in “red” areas with a high share of “iron-rich soils” and 4 out of 13 sites being on the boundary of HWSD mapping units with markedly different fractions of iron soils. Here, selection of study sites would have benefited from a sampling strategy based on soil maps. Apart from these sampling issues, the validation still yields a mixed picture. It correctly identifies two sites with known iron-toxicity risk (Gagnoa, Korhogo) and it correctly identifies four sites with low iron-toxicity risk (Abengourou, Boundiali, Soubré, Touba). But results are more ambiguous in three sites:

• in Bouna in the north-east, 33% of samples had medium iron-toxicity scores, while there is hardly any iron there according to HWSD;
• in Daloa in the centre of Côte d’Ivoire, 95% of observed fields had no visual iron-toxicity symptoms while according to HWSD the share of “iron-rich soils” in this place is around 50%;
• in Odienné in the north-west, 77% of the samples was free from iron-toxicity stress while according to HWSD the share of “iron-rich soils” in this place is around 50%.

As noted, HWSD also has a number of uncertainties, including the lack of georeferenced soil units within the larger soil mapping units. It is theoretically possible therefore that the iron-toxicity mapping is more accurate than I think, if samplings in Daloa and Odienné happened to take place in that share of the HWSD soil unit with no iron. It could also mean that the iron-toxicity mapping is too course, causing a certain amount of random error. Therefore, considerable uncertainty still exists in the mapping of areas prone to iron toxicity.

## Footnotes

1 The World Reference Base for Soil Resources (WRB) is the international standard taxonomic soil classification system endorsed by the International Union of Soil Sciences (IUSS). It was developed by an international collaboration coordinated by the International Soil Reference and Information Centre (ISRIC) and sponsored by the IUSS and FAO via its Land & Water Development division. It replaces the previous FAO soil classification. The HWSD dataset used in this paper is still based on the FAO soil classification (FAO et al., 2012). A description of the FAO90 soil types and their formation by geology, climatic and human factors in given by Driessen and Dudal (1989) and Dudal and Driessen (1991).

## References

[bib0005] Akinbile C.O., Adegbola O.A., Akande S.O. (2016). Determining the effect of salt-induced soil on paddy rice development and yield using GIS mapping. Paddy Water Environ..

[bib0010] Akpan A.E., Ebong E.D., Ekwok S.E. (2015). Assessment of the state of soils, shallow sediments and groundwater salinity in Abi Cross River State, Nigeria. Environ. Earth Sci..

[bib0015] Anderson W., You L., Wood S., Wood-Sichra U., Wu W. (2015). An analysis of methodological and spatial differences in global cropping systems models and maps. Glob. Ecol. Biogeogr..

[bib0020] Audebert A., Fofana M. (2009). Rice yield gap due to iron toxicity in West Africa. J. Agron. Crop Sci..

[bib0025] Audebert A., Narteh L.T., Kiepe P., Millar D., Beks B. (2006). Iron Toxicity in Rice-Based Systems in West Africa.

[bib0030] Balasubramanian V., Sie M., Hijmans R.J., Otsuka K., Sparks D.L. (2007). Increasing rice production in sub-Saharan Africa: challenges and opportunities. Advances in Agronomy.

[bib0035] Barbiero L., Cunnac S., Mane L., Laperrousaz C., Hammecker C., Maeght J.L. (2001). Salt distribution in the Senegal middle valley–analysis of a saline structure on planned irrigation schemes from N’Galenka creek. Agric. Water Manag..

[bib0040] Becker M., Asch F. (2005). Iron toxicity in rice-conditions and management concepts. J. Plant Nutr. Soil Sci..

[bib0045] Boling A.A., Bouman B.A.M., Tuong T.P., Murty M.V.R., Jatmiko S.Y. (2007). Modelling the effect of groundwater depth on yield-increasing interventions in rainfed lowland rice in Central Java, Indonesia. Agric. Syst..

[bib0050] Bouman B.A.M., Wopereis M.C.S., Kropff M.J., Tenberge H.F.M., Tuong T.P. (1994). Water-use efficiency of flooded rice fields. 2. Percolation and seepage losses. Agric. Water Manag..

[bib0055] Bouman B.A.M., Kropff M.J., Tuong T.P., Wopereis M.C.S., ten Berge H.F.M., van Laar H.H. (2001). ORYZA2000: Modeling Lowland Rice.

[bib0060] Bouman B.A.M., Humphreys E., Tuong T.P., Barker R. (2007). Rice and water. Adv. Agron..

[bib0065] Chérif M., Audebert A., Fofana M., Zouzou M. (2009). Evaluation of iron toxicity on lowland irrigated rice in West Africa. Tropicultura.

[bib0070] Danvi A., Jütten T., Giertz S., Zwart S.J., Diekkrüger B. (2016). A spatially explicit approach to assess the suitability for rice cultivation in an inland valley in central Benin. Agric. Water Manag..

[bib0075] de Vries M.E., Leffelaar P.A., Sakane N., Bado B.V., Giller K.E. (2011). Adaptability of irrigated rice to temperature change in Sahelian environments. Exp. Agric..

[bib0080] Diagne A., Amovin-Assagba E., Futakuchi K., Wopereis M.C.S., Wopereis M.C.S., Johnson D.E., Ahmadi N., Tollens E., Jalloh A. (2013). Estimation of cultivated area, number of farming households and yield for major rice-growing environments in Africa. Realizing Africa’s Rice Promise.

[bib0085] Diagne A., Amovin-Assagba E., Futakuchi K., Wopereis M.C.S., Wopereis M.C.S., Johnson D.E., Ahmadi N., Tollens E., Jalloh A. (2013). Farmers perceptions of the biophysical constraints to rice production in sub-Saharan Africa, and potential impact of research. Realizing Africa’s Rice Promise.

[bib0090] Dingkuhn M., Radanielina T., Raboin L.M., Dusserre J., Ramantsoanirina A., Sow A., Manneh B., Balde A.B., Soulié J.C., Shrestha S., Ahmadi N., Courtois B. (2015). Field phenomics for response of a rice diversity panel to ten environments in Senegal and Madagascar. 2. Chilling-induced spikelet sterility. Field Crop Res..

[bib0095] Dingkuhn M., Pasco R., Pasuquin J.M., Damo J., Soulie J.C., Raboin L.M., Dusserre J., Sow A., Manneh B., Shrestha S., Kretzschmar T. (2017). Crop-model assisted phenomics and genome-wide association study for climate adaptation of indica rice. 2. Thermal stress and spikelet sterility. J. Exp. Bot..

[bib0100] Driessen P.M., Dudal R. (1989). Lecture Notes on the Geography, Formation, Properties and Use of the Major Soils of the World.

[bib0105] Dudal R., Driessen P.M. (1991). The Major Soils of the World: Lecture Notes on Their Geography, Formation, Properties and Use.

[bib0110] El-Shahway A.S., Mahmoud M.M.A., Udeigwe T.K. (2016). Alterations in soil chemical properties induced by continuous rice cultivation: a study on the Arid Nile Delta Soils of Egypt. Land Degrad. Dev..

[bib0115] FAO, IIASA, ISRIC, ISSCAS, JRC (2012). Harmonized World Soil Database (version 1.2).

[bib0120] Fischer G., Nachtergaele F.O., Prieler S., Teixeira E., Tóth G., van Velthuizen H., Verelst L., Wiberg D. (2013). Global Agro-Ecological Zones (GAEZ) Version 3.0.

[bib0125] Genon J.G., Dehepcee N., Duffy J.E., Delvaux B., Hennebert P.A. (1994). Iron toxicity and other chemical soil constraints to rice in Highland Swamps of Burundi. Plant Soil.

[bib0130] Grattan S.R., Zeng L., Shannon M.C., Roberts S.R. (2002). Rice is more sensitive to salinity than previously thought. Calif. Agric..

[bib0135] Gupta S.K., Sharma S.K. (1990). Response of crops to high exchangeable sodium percentage. Irrig. Sci..

[bib0140] Haefele S.M., Nelson A., Hijmans R.J. (2014). Soil quality and constraints in global rice production. Geoderma.

[bib0145] Heinemann A.B., Barrios-Perez C., Ramirez-Villegas J., Arango-Londoño D., Bonilla-Findji O., Medeiros J.C., Jarvis A. (2015). Variation and impact of drought-stress patterns across upland rice target population of environments in Brazil. J. Exp. Bot..

[bib0150] Hengl T., Roudier P., Beaudette D., Pebesma E. (2015). Plotkml: scientific visualization of spatio-temporal data. J. Stat. Softw..

[bib0155] Howeler R.H. (1973). Iron-induced oranging disease of rice in relation to physicochemical changes in a flooded oxisol. Soil Sci. Soc. Am. J..

[bib0160] Jugsujinda A., Patrick W.H. (1993). Evaluation of toxic conditions associated with oranging symptoms of rice in a flooded Oxisol in Sumatra, Indonesia. Plant Soil.

[bib0165] Kashenge-Killenga S., Meliyo J., Urassa G., Kongo V. (2016). Extent of salt-affected soils and their effects in irrigated and lowland rain-fed rice growing areas of Southwestern Tanzania. Climate Change and Multi-Dimensional Sustainability in African Agriculture: Climate Change and Sustainability in Agriculture.

[bib0170] Kotb T.H.S., Watanabe T., Ogino Y., Tanji K.K. (2000). Soil salinization in the Nile Delta and related policy issues in Egypt. Agric. Water Manag..

[bib0175] Kottek M., Grieser J., Beck C., Rudolf B., Rubel F. (2006). World map of the Köppen-Geiger climate classification updated. Meteorol. Z..

[bib0180] Laborte A.G., Gutierrez M.A., Balanza J.G., Saito K., Zwart S.J., Boschetti M., Murty M.R.V., Villano L., Aunario J.K., Reinke R., Koo J., Hijmans R.J., Nelson A. (2017). RiceAtlas, a spatial database of global rice calendars and production. Nat.: Sci. Data.

[bib0185] Menete M.Z.L., van Es H.M., Brito R.M.L., DeGloria S.D., Famba S. (2008). Evaluation of system of rice intensification (SRI) component practices and their synergies on salt-affected soils. Field Crop Res..

[bib0190] Pavelic P., Villholth K.G., Shu Y., Rebelo L.M., Smakhtin V. (2013). Smallholder groundwater irrigation in Sub-Saharan Africa: country-level estimates of development potential. Water Int..

[bib0195] Portmann F.T., Siebert S., Doll P. (2010). MIRCA2000-global monthly irrigated and rainfed crop areas around the year 2000: a new high-resolution data set for agricultural and hydrological modeling. Glob. Biogeochem. Cycles.

[bib0200] Prade K., Ottow J.C.G., Jacq V.A. (1993). Excessive Iron Uptake (Iron Toxicity) by Wetland Rice (Oryza sativa L.) on an Acid Sulphate Soil in the Casamance/Senegal.

[bib0205] Rodenburg J., Demont M., Zwart S.J., Bastiaans L. (2016). Parasitic weed incidence and related economic losses in rice in Africa. Agric. Ecosyst. Environ..

[bib0210] Ruane A.C., Goldberg R., Chryssanthacopoulos J. (2015). Climate forcing datasets for agricultural modeling: merged products for gap-filling and historical climate series estimation. Agric. For. Meteorol..

[bib0215] Sahrawat K.L. (2004). Iron toxicity in wetland rice and the role of other nutrients. J. Plant Nutr..

[bib0220] Sakané N., Alvarez M., Becker M., Böhme B., Handa C., Kamiri H.W., Langensiepen M., Menz G., Misana S., Mogha N.G., Möseler B.M., Mwita E.J., Oyieke H.A., van Wijk M.T. (2011). Classification, characterisation, and use of small wetlands in East Africa. Wetlands.

[bib0225] Schmitter P., Zwart S.J., Danvi A., Gbaguidi F. (2015). Contributions of lateral flow and groundwater to the spatio-temporal variation of irrigated rice yields and water productivity in a West-African inland valley. Agric. Water Manag..

[bib0230] Sikirou M., Saito K., Achigan-Dako E.G., Drame K.N., Ahanchede A., Venuprasad R. (2015). Genetic improvement of iron toxicity tolerance in rice-progress, challenges and prospects in West Africa. Plant Prod. Sci..

[bib0235] Sikirou M., Saito K., Drame K.N., Saidou A., Dieng I., Ahanchede A., Venuprasad R. (2016). Soil-based screening for iron toxicity tolerance in rice using pots. Plant Prod. Sci..

[bib0240] Sylla M., Stein A., Vanbreemen N., Fresco L.O. (1995). Spatial variability of soil-salinity at different scales in the mangrove rice agroecosystem in West-Africa. Agric. Ecosyst. Environ..

[bib0245] Tanji K.K., Kielen N.C. (2002). Agricultural Drainage Water Management in Arid and Semi-Arid Areas.

[bib0250] Thiam E.H.I., Singh V.P. (1998). Spatial and temporal variability of salinity in Casamance River Basin, Southern Senegal, West Africa. Hydrol. Process..

[bib0255] van Asten P.J.A., Barbiero L., Wopereis M.C.S., Maeght J.L., van der Zee S.E.A.T.M. (2003). Actual and potential salt-related soil degradation in an irrigated rice scheme in the Sahelian zone of Mauritania. Agric. Water Manag..

[bib0260] van Asten P.J.A., Wopereis M.C.S., Haefele S., Ould Isselmou M., Kropff M.J. (2003). Explaining yield gaps on farmer-identified degraded and non-degraded soils in a Sahelian irrigated rice scheme. Neth. J. Agric. Sci..

[bib0265] van Asten P.J.A., Barro S.E., Wopereis M.C.S., Defoer T. (2004). Using farmer knowledge to combat low productive spots in rice fields of a Sahelian irrigation scheme. Land Degrad. Dev..

[bib0270] van Diepen C.A., Wolf J., van Keulen H., Rappoldt C. (1989). WOFOST – a simulation-model of crop production. Soil Use Manag..

[bib0275] van Genuchten M.T. (1980). Closed-form equation for predicting the hydraulic conductivity of unsaturated soils. Soil Sci. Soc. Am. J..

[bib0280] van Ittersum M.K., van Bussel L.G.J., Wolf J., Grassini P., van Wart J., Guilpart N., Claessens L., De Groot H., Wiebe K., Mason-D’Croz D., Yang H., Boogaard H., van Oort P.A.J., van Loon M.P., Saito K., Adimo O., Adjei-Nsiah S., Agali A., Bala A., Chikowo R., Kaizzi K., Kouressy M., Makoi J.H.J.R., Ouattara K., Tesfaye K., Cassman K.G. (2016). Can sub-Saharan Africa feed itself?. Proc. Natl. Acad. Sci. U. S. A..

[bib0285] van Oort P.A.J., Zhang T.Y., de Vries M.E., Heinemann A.B., Meinke H. (2011). Correlation between temperature and phenology prediction error in rice (Oryza sativa L.). Agric. For. Meteorol..

[bib0290] van Oort P.A.J., De Vries M.E., Yoshida H., Saito K. (2015). Improved climate risk simulations for rice in arid environments. PLoS One.

[bib0295] van Oort P.A.J., Saito K., Tanaka A., Amovin-Assagba E., Van Bussel L.G.J., van Wart J., de Groot H., van Ittersum M.K., Cassman K.G., Wopereis M.C.S. (2015). Assessment of rice self-sufficiency in 2025 in eight African countries. Glob. Food Secur..

[bib0300] van Oort P.A.J., Balde A., Diagne M., Dingkuhn M., Manneh B., Muller B., Sow A., Stuerz S. (2016). Intensification of an irrigated rice system in Senegal: crop rotations, climate risks, sowing dates and varietal adaptation options. Eur. J. Agron..

[bib0305] Van Oort P.A.J., Zwart S.J. (2018). Impacts of climate change on rice production in Africa and causes of simulated yield changes. Glob. Change Biol..

[bib0310] Van Wart J., van Bussel L.G.J., Wolf J., Licker R., Grassini P., Nelson A., Boogaard H., Gerber J., Mueller N.D., Claessens L., van Ittersum M.K., Cassman K.G. (2013). Use of agro-climatic zones to upscale simulated crop yield potential. Field Crop Res..

[bib0315] Vergara B.S., Chang T.T. (1985). The Flowering Response of the Rice Plant to Photoperiod. A Review of Literature.

[bib0320] Villholth K.G. (2013). Groundwater irrigation for smallholders in Sub-Saharan Africa–a synthesis of current knowledge to guide sustainable outcomes. Water Int..

[bib0325] Waddington S.R., Li X., Dixon J., Hyman G., de Vicente M.C. (2010). Getting the focus right: production constraints for six major food crops in Asian and African farming systems. Food Secur..

[bib0330] Windmeijer P.N., Andriesse W.E. (1993). Inland Valleys in West Africa: An Agro-Ecological Characterization of Rice-Growing Environments.

[bib0335] Wolanski E., Cassagne B. (2000). Salinity intrusion and rice farming in the mangrove-fringed Konkoure River delta, Guinea. Wetl. Ecol. Manag..

[bib0340] Wolf J., Ouattara K., Supit I. (2015). Sowing rules for estimating rainfed yield potential of sorghum and maize in Burkina Faso. Agric. For. Meteorol..

[bib0345] Wopereis M.C.S., Bouman B.A.M., Kropff M.J., Tenberge H.F.M., Maligaya A.R. (1994). Water-use efficiency of flooded rice fields. 1. Validation of the soil-water balance model Sawah. Agric. Water Manag..

[bib0350] Yoshida H., Horie T., Shiraiwa T. (2006). A model explaining genotypic and environmental variation of rice spikelet number per unit area measured by cross-locational experiments in Asia. Field Crop Res..

[bib0355] You L., Wood-Sichra U., Fritz S., Guo Z., See L., Koo J. (2014). Spatial Production Allocation Model (SPAM) 2005 Beta Version. http://mapspam.info.

[bib0360] You L., Wood S., Wood-Sichra U., Wu W. (2014). Generating global crop distribution maps: from census to grid. Agric. Syst..

[bib0365] Young A. (1999). Is there really spare land? A critique of estimates of available cultivable land in developing countries. Environ. Dev. Sustain..

[bib0370] Zhang T., Li T., Yang X., Simelton E. (2016). Model biases in rice phenology under warmer climates. Sci. Rep..

